# Understanding nanoparticle endocytosis to improve targeting strategies in nanomedicine

**DOI:** 10.1039/d0cs01127d

**Published:** 2021-03-05

**Authors:** Mauro Sousa de Almeida, Eva Susnik, Barbara Drasler, Patricia Taladriz-Blanco, Alke Petri-Fink, Barbara Rothen-Rutishauser

**Affiliations:** Adolphe Merkle Institute, University of Fribourg Chemin des Verdiers 4 1700 Fribourg Switzerland barbara.rothen@unifr.ch; Department of Chemistry, University of Fribourg Chemin du Musée 9 1700 Fribourg Switzerland

## Abstract

Nanoparticles (NPs) have attracted considerable attention in various fields, such as cosmetics, the food industry, material design, and nanomedicine. In particular, the fast-moving field of nanomedicine takes advantage of features of NPs for the detection and treatment of different types of cancer, fibrosis, inflammation, arthritis as well as neurodegenerative and gastrointestinal diseases. To this end, a detailed understanding of the NP uptake mechanisms by cells and intracellular localization is essential for safe and efficient therapeutic applications. In the first part of this review, we describe the several endocytic pathways involved in the internalization of NPs and we discuss the impact of the physicochemical properties of NPs on this process. In addition, the potential challenges of using various inhibitors, endocytic markers and genetic approaches to study endocytosis are addressed along with the principal (semi) quantification methods of NP uptake. The second part focuses on synthetic and bio-inspired substances, which can stimulate or decrease the cellular uptake of NPs. This approach could be interesting in nanomedicine where a high accumulation of drugs in the target cells is desirable and clearance by immune cells is to be avoided. This review contributes to an improved understanding of NP endocytic pathways and reveals potential substances, which can be used in nanomedicine to improve NP delivery.

## Abstract 2

This review contributes to the current understanding of NPs cellular uptake and gives an overview about molecules, which can enhance or decrease cellular internalization of NPs.

## Graphical abstract

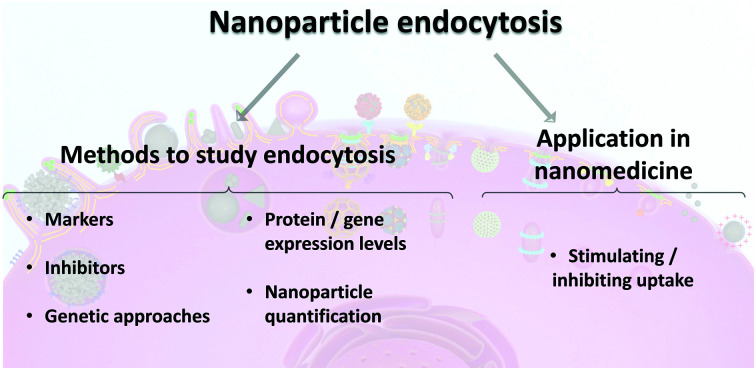

## 1. Introduction

Nanotechnology is a multidisciplinary field comprising, among others, chemistry, physics, biology, and medicine, which focuses on the design, production, and application of nanosized systems (*e.g.*, devices, paints, food additives, and drug delivery systems).^1^ First discussions about nanotechnology date back to 1959, when Richard Feynman mentioned the opportunity of controlling atom by atom the production of miniaturized machines.^2,3^

The term “nanotechnology” was introduced only a couple of years later, in 1974, by Norio Taniguchi.^4^ The possibility to manipulate materials at nanoscale provided a boost in the development of novel materials with different performance in comparison to the bulk material.^5^ To date, there are different definitions of nanomaterials, depending on the application field.^6,7^

The International Organization for Standardization (ISO)^8^ defines a nanomaterial as a “material with any external dimension in the nanoscale or having an internal structure or surface structure in the nanoscale (1–100 nm)”. Similarly, in 2011, the European Commission^9^ adopted a definition for a nanomaterial: “A natural, incidental or manufactured material containing particles, in an unbound state or as an aggregate or as an agglomerate and where, for 50% or more of the particles in the number size distribution, one or more external dimensions is in the size range 1–100 nm”. The United States Food and Drug administration (US FDA)^6^ states that nanomaterials are “materials up to one micron if these ones exhibit properties or phenomena that are attributable to its dimensions”. In this review, a size range between 1–1000 nm is considered for nanomaterials and nanoparticles (NPs). The NP dimensions mentioned throughout the manuscript are expressed as a diameter, unless otherwise stated.

Pharmaceutical, medical, and device manufacturing industries are developing new nanomaterials for biomedical applications, such as drug delivery systems, biosensors and medical nanodevices.^9,10^ For drug delivery applications, cellular uptake, intracellular fate, and accumulation of NPs loaded with therapeutics play an important role in successful disease treatment.^11^ Several factors should be considered in the development of safe and efficient NP systems for medical purposes (*i.e.*, nanomedicine): (i) NPs physico-chemical properties (*e.g.*, size, shape, surface charge, and stiffness); (ii) colloidal stability; (iii) degradation rate, *i.e.* solubility; (iv) biocompatibility; (v) bioaccumulation; (vi) route of administration (*e.g.*, intravenous, oral, inhalation and dermal) and (vii) target cell/tissue. The principal process used by cells to internalize NPs is endocytosis.^12^ Endocytosis is an important mechanism for cellular uptake of nutrients, regulation of cell surface receptors, control of cell polarity, motility, and signaling cascades.^12,13^

A thorough understanding of endocytosis is essential to optimize the safe-by-design, cellular targeting and uptake of NPs. It is still challenging to generalize the current findings, due to the variation on the endocytic mechanisms dependent on cell types and for different NPs.^14–17^ In addition, when NPs interact with physiological fluids such as mucus, lymph fluid, or blood, they can interact with different biomolecules including opsonins that promote cellular recognition and clearance by the mononuclear phagocyte system (MPS).^18^ Also the presence of efflux pumps, overexpression of specific transporters on the cell membrane, as well as mitosis can reduce NP accumulation in the target cells.^19,20^ Consequently, a very low number of NPs reach the target cells and might not be sufficient to treat the disease.^21^ For this reason, it becomes important to enhance NP-based targeted delivery and, at the same time, avoid internalization by MPS if this system is not the targeted one. Various stimuli, such as inflammatory cytokines,^22^ multiple NP co-exposure,^23,24^ or functionalization with ligands^25–27^ were described to increase NP uptake in target cells. Several other substances have also been found to decrease NP internalization.^28,29^ This effect is desired to avoid NP accumulation in non-target cells or organs, where they could cause unintended acute or chronic toxicity.^21^

This review contributes to the current understanding of NP cellular uptake and gives an overview about molecules, which can enhance or decrease cellular internalization of NPs. A description of the different endocytic mechanisms is included together with the approaches for NP quantification.

## 2. Cellular uptake mechanisms of NPs

NPs may enter the body *via* inhalation, oral ingestion, dermal and ocular penetration, and injection (intravenous, intramuscular and subcutaneous).^21,30^ Upon contact with physiological fluids or physical barriers, *via* different administration routes, NPs may undergo a process of aggregation and/or dissolution.^31,32^ In addition, the different constitution of physiological fluids (*e.g.*, proteins, lipids and electrolytes) including blood, respiratory and gastrointestinal mucus, and tear fluid might affect NP properties and thus cell–NP interactions.^33–35^

Inhalation has been used for the administration of nanomaterials to treat lung diseases or for systemic delivery.^36^ The lung possesses a large internal surface area (around 150 m^2^) and NPs can deposit in alveoli as opposed to their bulk counterparts, which is an advantage for pulmonary delivery.^37,38^ Furthermore, they might reach the peripheral gas exchange region and pass the air-blood tissue barrier to enter systemic circulation.^39^ Only a small amount of inhaled NPs reach systemic circulation because a large fraction of NPs is cleared by macrophages and the translocation of NPs inversely depends on particle size.^40,41^ In intravenous injection, NPs tend to penetrate and accumulate within the leaky tumor vasculature, which is designated as an enhanced permeation and retention (EPR) effect.^30^ A large retention of NPs by the reticuloendothelial system (RES), such as the liver, kidneys and spleen is observed when NPs are administered intravenously.^42^ The skin is the largest human organ, acting as an effective first barrier against external factors, such as pathogens.^43^ In dermal applications NPs usually remain on the skin surface, however for transdermal applications NPs may be able to penetrate into the stratum corneum *via* intercellular pathways, *e.g.* hair follicles or glandular tissue, or permeate the whole stratum corneum into deeper skin layers.^44,45^ The topical ocular drug delivery, usually based on eye drops, is used to treat ocular disorders.^46^ This approach usually requires the interaction of the drug with the sclera and the different tissues of the anterior segment (*e.g.*, cornea, iris and conjunctiva).^46^ NPs have been studied as delivery systems for ophthalmic drugs and have revealed increased corneal permeability.^47^ The drug bioavailability in the target tissue may be affected by precorneal factors and anatomical barriers such as nasolacrimal drainage, tear turnover, and blinking.^46^

**Fig. 1:**
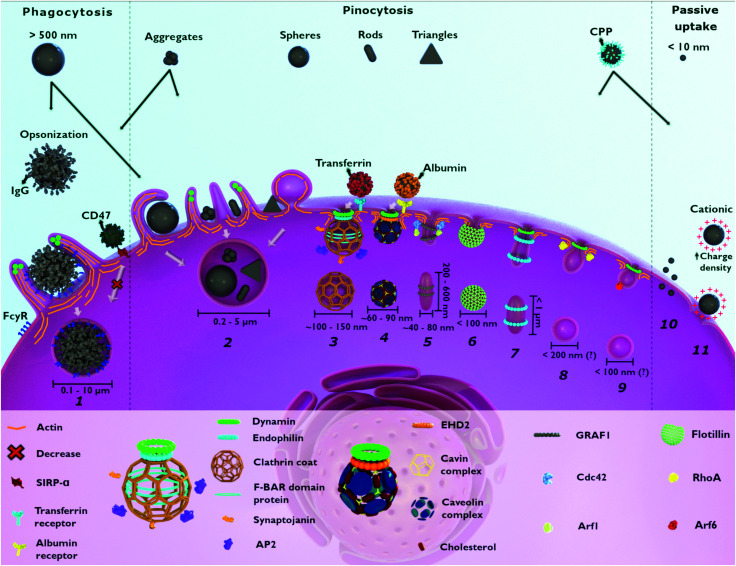
Possible entry mechanisms for nanoparticles (NPs). Large NPs (>500 nm) and aggregates enter the cell through phagocytosis (1) and macropinocytosis (2). NP opsonization *via* IgG leads to cellular recognition (FcγR) in phagocytes. Pinocytosis includes different mechanisms: macropinocytosis (2), clathrin-mediated endocytosis (3), caveolae-dependent endocytosis (4), CLIC–GEEC (5), flotillin-assisted endocytosis (6), fast-endophilin-mediated endocytosis (7), RhoA-dependent endocytosis (8) and Arf-6-associated endocytosis (9). As a non-selective endocytic process, macropinocytosis (2) is associated with the internalization of different NPs. Smaller NPs (<10 nm) and cationic NPs, with high charge density, enter the cell *via* direct penetration (10) and pore formation (11), respectively. NPs surface functionalization with different molecules has an impact on cellular uptake. NPs functionalized with transferrin and albumin are taken up through clathrin-mediated (3) and caveolae-dependent endocytosis (4), respectively. NPs functionalized with CPP can be internalized passively (10 and 11) as well as *via* other endocytic pathways. The functionalization of NPs with CD47 reduces phagocytosis (1). Flotillin-assisted endocytosis (6) and Arf-6-associated endocytosis (9) can occur both, in the presence or absence of dynamin. CPP: cell penetrating peptide. IgG: immunoglobulin G. CD47: cluster of differentiation 47. FcγR: Fc gamma receptor. SIRPα: signal regulatory protein alpha. AP2: adaptor protein 2. EHD2: Eps15-homology domain containing protein 2. GRAF1: GTPase regulator associated with focal adhesion kinase-1. CDC42: cell division cycle 42. Arf1: ADP-ribosylation factor 1. RhoA: Ras homolog family member A. Arf6: ADP-ribosylation factor 6. (?) – vesicle size still unclear.

Following this first interplay with human chemical and physical barriers, NPs come in contact with the outer cell membrane. Herein, they may interact with the components of the outer plasma membrane and be subsequently internalized.^48^ The main cellular process of NP internalization is endocytosis and involves the invagination/ruffling of the cell membrane followed by the formation of intracellular/endocytic vesicles (Fig. 1).^17^ Depending on the cell–NP interaction, different signaling cascades can occur leading to various structural changes at the cell surface.^49^ These processes, together with the vast diversity of molecules (*e.g.*, surface receptors, membrane lipids, cargo and adaptor proteins) needed for efficient endocytic trafficking of NPs, enable endocytosis to be categorized into two main types: phagocytosis and pinocytosis.^50,51^ Phagocytosis encompasses the uptake of large particles (≥0.5 μm) and is only performed by specialized cells, as described below (Section 2.1).^52^ Pinocytosis is associated with fluid-phase uptake and, based on the majority of the literature, includes macropinocytosis, clathrin-mediated endocytosis, caveolin-mediated endocytosis and clathrin/caveolae-independent endocytosis.^50,53^ These mechanisms occur in almost every eukaryotic cell.^13^

In this section, the mechanisms and the influence of the physicochemical properties of NPs on the subsequent uptake will be discussed. A common feature in endocytosis is the localization of NPs into endocytic vesicles after internalization. In addition, the occurrence of other internalization routes that do not involve vesicle formation, such as passive diffusion and pore formation by cell membrane disruption, will be discussed as well.^11^

### 2.1 Phagocytosis

Phagocytosis is an endocytic route carried out by professional phagocytes *i.e.*, macrophages, monocytes, dendritic cells, osteoclasts, eosinophils and neutrophils where foreign bodies such as bacteria or fungi and cell debris are ingested and eliminated.^54^ In addition, it was shown that nonprofessional phagocytes such as fibroblasts, epithelial and endothelial cells, also possess phagocytic activity, but only to a limited extent, not being able to completely eliminate microorganisms.^55,56^

Phagocytosis is usually associated with the uptake of large particles. In contrast, several studies also reported the phagocytosis of nanometer-sized particles, such as gold,^57,58^ silver,^59^ and polymeric NPs.^60^ It is hypothesized that this occurs mainly for aggregated NPs after an opsonization process (Fig. 1).^61,62^ Once NPs are dispersed in a physiological fluid such as mucosal, lymph fluid, or blood, they will interact with different proteins that will adsorb at their surface, creating the so-called “protein corona”.^34,63^ The immunoglobulins and complement proteins present in the protein corona, namely as opsonins, are recognized by opsonic receptors, *i.e.*, Fc receptors (FcR) and complement receptors leading to NP internalization.^54,64^ Nevertheless, phagocytes also express non-opsonic receptors (*e.g.*, mannose and scavenger receptors) that are able to interact directly with the molecular groups on the NP surface.^64,389^ For efficient recognition of the NPs, cooperation between multiple phagocyte receptors can occur depending on the density of both the molecules on the NPs surface and the receptors at the cellular membrane.^56^ The relative mobility of the receptors on the cell membrane and their affinity for the NPs influence phagocytosis efficiency.^65^

The protein composition and conformation on the NPs surface influence the interaction and recognition by the phagocyte surface receptors.^33^ The receptors involved in this recognition dictate the subsequent signaling cascade and may potentially initiate inflammatory events (*e.g.*, FcR).^62^ Silica (SiO_2_) NPs of 50 and 100 nm triggered inflammation in THP-1 macrophage-like cells by the activation of the scavenger receptor A1, while 10 and 1000 nm did not.^66^ In a different context, a pre-coating of SiO_2_ NPs and single-carbon nanotubes coated with the surfactant Pluronic F127 reduced the adsorption of serum proteins and inhibited the anti-inflammatory effect on murine macrophages (RAW 264.7 cells).^67^ Therefore, the proteins adsorbed on the NP surface and the following receptor activation affect both the uptake of NPs and cell reactivity (*i.e.* inflammatory response).

Receptor-mediated phagocytosis is initiated upon cell–NP interaction, which leads to a signaling cascade resulting in the polymerization of actin filaments, membrane cup-shaped extensions and subsequent internalization of NPs.^56^ The formed phagosome containing NPs matures by a series of changes in its membrane composition and content. At the end it fuses with a lysosome, an acidic vesicle, that, depending on the NP material, is able to digest the ingested NPs.^68^

### 2.2 Macropinocytosis

Similar to phagocytosis, macropinocytosis is an actin-dependent process involved in the engulfment of fluids and micron-sized particles.^69^ This mechanism is a non-selective process where plasma membrane ruffles engulf high amounts of an external fluid, including particles and dissolved molecules, into large vesicles called macropinosomes (0.2–5 μm).^70^ Macropinocytosis allows the internalization of larger macromolecules by cells that do not possess phagocytic activity, which would not be possible through other endocytic mechanisms such as clathrin- and caveolae-mediated endocytosis.^69^ Depending on the cell type, macropinocytosis can occur in a constitutive or inducible way.^71^ In response to the stimulation by growth factors (*e.g.*, epidermal and platelet-derived growth factors) and other molecules, actin-rich extensions of the plasma membrane, referred to as ruffles, can retreat back into the cell membrane or curve into circular ruffles that undergo membrane fission to form macropinosomes.^72^ This process, depending on the activation stimulus, may involve many molecules important to actin polymerization, cytoskeleton organization, macropinosome formation and closure. Here are encompassed several small GTPases from the Ras superfamily, kinases (p21-activated kinase 1 and protein kinase C) and lipids (phosphoinositides and diacylglycerol).^73^ More details about the molecular machinery involved in this process are available in other reviews.^71,72,74^

The uptake of NPs through macropinocytosis occurs in a non-specific way, meaning that NPs are internalized due to the close contact with the plasma membrane where the ruffle formation starts.^75^ Therefore, the uptake through this mechanism is usually not dependent on specific NP properties such as size or shape. It is possible that adsorbed proteins on the NP surface or NPs functionalized with specific molecules are able to stimulate macropinocytosis, as will be discussed later in this review.

**Fig. 2:**
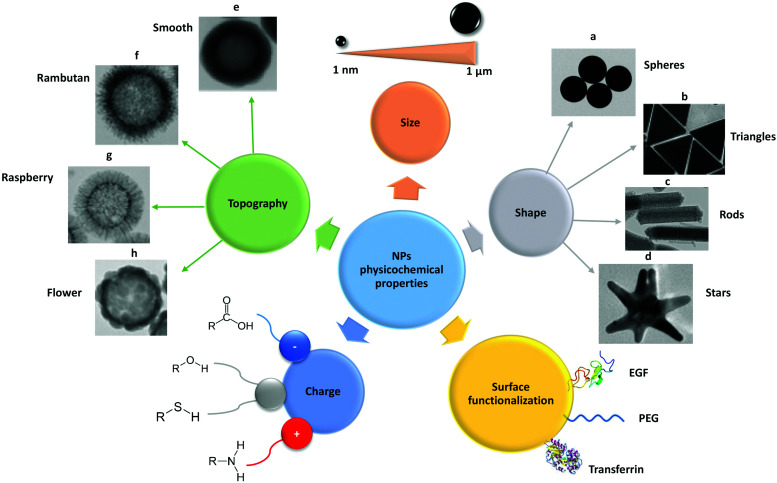
Schematic representation of the main physico-chemical properties of NPs, such as size, shape, surface functionalization, charge and topography. These properties need to be considered when employing NPs as carriers for drug delivery in nanomedicine. EGF – epidermal growth factor; PEG – polyethylene glycol. Adapted with permission from (a) ref. 421, (b) ref. 422, (c) ref. 423, (d) ref. 424 and (e–h) ref. 154. Copyright (a) Susnik *et al.* 2020, (b) Kuttner *et al.* 2018, (c) Madathiparambil Visalakshan *et al.* 2020, (d) Liu *et al.* 2015 and (e–h) Singh *et al.* 2019.

### 2.3 Clathrin-mediated endocytosis (CME)

CME is the most studied endocytic mechanism and all the molecular details and cargo specificity are well described in the literature.^50,76,77^ It is considered as one of the most important mechanisms for NP uptake^17^ but is also related to many other functions such as regulation of expression of surface proteins and uptake of nutrients including iron *via* the transferrin receptor.^11^ The designation “clathrin-mediated endocytosis” is associated with the most abundant protein in the process, the triskelion clathrin that assembles in hexagons and pentagons to form a lattice-like coat around the endocytic vesicles.^78^ The process involving the formation of clathrin-coated vesicles (CCVs) can be divided into five different steps: initiation, cargo selection, coat assembly, scission, and un-coating.^76^ All these processes involve a set of proteins that localize intracellularly and are recruited to the plasma membrane in a coordinated manner.^77^ The protein machinery includes: (i) F-BAR domain-containing proteins important to initialize membrane curvature; (ii) clathrin adaptor proteins such as AP2, synaptojanin, and other accessory proteins – AP180, epsin and SNX9 – associated with cargo recognition, coat assembly and stabilization of membrane curvature; (iii) GTPase dynamin and endophilin for vesicle scission.^76,79^ For several years this mechanism was also designated as receptor-mediated endocytosis (RME), because it was believed that only specific interactions between the cargo and the receptor could lead to CME.^11,17^ Internalization of non-specific cargoes can also occur *via* non-specific interactions, such as hydrophobic and/or electrostatic interactions.^11^ Transferrin and epidermal growth factor (EGF) are ligands that bind to their specific receptors leading to RME *via* clathrin.^80^

The CCV can have different sizes in different cell types depending on the cargo.^13,77^ During chemical synapses where fast processes are required, CCVs are usually smaller (∼50 nm), compared to CCVs in epithelial cells with a size of ∼120–150 nm.^13^ Nevertheless, the maximum reported size for CCVs was 200 nm.^76^ Therefore, the size range of CCVs and the occurrence of this process in all eukaryotic cells are the reason why CME is one of the principal mechanisms for NP uptake.

Several studies investigated the uptake of NPs in different cell types *via* CME. The route of internalization of 40 nm polystyrene (PS) NPs was studied in different cell types, cervical epithelial (HeLa), lung epithelial (A549), brain astrocytoma and macrophage (J774A.1), revealing the involvement of CME in all cells in combination with other mechanisms.^60,81^ In addition, several other NPs have been shown to be taken up by cells through CME, including fluorescein isothiocyanate (FITC)-incorporated silica-coated core-shell superparamagnetic iron oxide (SPION@SiO_2_),^82^ AuNPs^83^ and poly(ethylene glycol)-d,l-polylactide (PEG-PLA) NPs.^84^ NPs taken up *via* CME usually end up in the acidic environment of lysosomes.^50^

### 2.4 Caveolae-dependent endocytosis

Together with CME caveolae-dependent endocytosis is a common route of internalization of NPs.^16^ Caveola is the designation for the flask-shaped invaginations with 50–100 nm that can be found in the plasma membrane of specific mammalian cells.^85^ These structures are abundant in smooth muscle, endothelia and adipocytes, covering approximately a third of the plasma membrane area.^86^ Two important components are essential for caveolae formation: caveolin-1, or caveolin-3 in striated muscle cells, and cavin 1.^87^ Other components such as caveolin-2, and cavins 2, 3 and 4, and different accessory proteins (*e.g.*, Eps-15 homology domain 2 (EHD2), PACSIN2 and dynamin 2) are also important for the formation, stabilization and scission of caveolae.^87–89^ Different cargoes have been linked to this pathway, including shiga and cholera bacterial toxins, non-enveloped viruses polyomavirus and simian virus 40, albumin, folic acid and plasma membrane components such as glycosylphosphatidylinositol (GPI)-anchored proteins.^87,88^ A commercially available NP conjugate of paclitaxel to human albumin (Nab-paclitaxel/Abraxane®) is successfully internalized by the cells through caveolae-mediated endocytosis.^90^ Similarly, lipid NPs with poly(styrene sulfonate) surface were revealed to be internalized *via* caveolae in HeLa and human endothelial cells (HUVEC).^91^

Caveolae also play other important roles including their involvement in transcytosis across endothelial cells allowing the transport of solutes between the blood and the peripheral tissues (*e.g.*, heart and lungs).^92,93^ An efficient transcytosis mechanism was observed for albumin-coated polymeric NPs of 20, 40 and 100 nm in lung endothelial cells (BLMVEC).^94^

### 2.5 Clathrin/caveolae-independent endocytosis

Besides the clathrin and caveolae-mediated endocytosis, which involve specific coated vesicles, NPs and other cargos such as cellular fluids, growth hormones and toxins, can be internalized *via* clathrin/caveolae-independent endocytosis.^95^ These mechanisms are usually cholesterol-dependent and require specific lipid composition.^11^ Despite the study of these processes, their contribution to endocytic uptake in mammalian cells is still not fully explained. Therefore, several ways of categorizing clathrin-/caveolae-independent endocytosis mechanisms are proposed in the literature.^96–98^ Herein, we have decided to categorize them according to the GTPases and associated proteins that are involved in the cellular entry pathway. In this regard, the clathrin/caveolae-independent endocytosis can be subdivided to: (i) clathrin-independent carriers/GPI-AP enriched early endosomal compartment (CLIC–GEEC), (ii) RhoA-dependent, (iii) Arf6-associated, (iv) flotillin-assisted and (v) fast endophilin-mediated endocytosis (for specific details see other publications^95,97,99–101^).

(i) The CLIC–GEEC pathway is a dynamin-independent process leading to the formation of tubular/ring-like invaginations of the plasma membrane of around 200–600 nm in length and 40–80 nm in width.^102^ This process principally involves two small GTPases, Cdc42 and Arf1, the multidomain protein GTPase regulator associated with focal adhesion kinase (GRAF1), cholesterol and actin.^95^ The principal cargoes internalized through this pathway are extracellular fluid, GPI-anchored proteins and cholera toxin B.^99^

(ii) Contrary to CLIC–GEEC, RhoA-dependent endocytosis is a dynamin-dependent process responsible for the internalization of the interleukin-2 receptor and *Clostridium botulinum* C2 toxin.^61^ RhoA, one of the Ras homologous (Rho) protein family of GTPases, is the principal small GTPase protein involved in this process.^103^ Additionally, several other molecules, including small GTPase Rac1, its downstream p21-activated kinases (*i.e.*, PAK-1 and PAK-2), and phosphatidylinositol 3-kinase are important regulators in this pathway.^104^

(iii) Although Arf6 was thought to be involved in macropinocytosis, the internalization of the major histocompatibility complex class I, β1-integrin and E-cadherin has been associated with Arf6-associated endocytosis in a clathrin- and dynamin-independent process.^101^ Uptake of other cargoes, including coxsackievirus A9 and the green fluorescent protein fused with the herpes simplex virus protein VP22, were shown to be dynamin-dependent.^105,106^ Arf6's key role is not in the process of endocytosis, but in recycling, and is thus referred to as Arf6-associated endocytosis.^107^

(iv) Flotillin-assisted endocytosis is another process that encompasses flotillin-1 (*i.e.*, reggie-2) and flotillin-2 (*i.e.*, reggie-1).^95^ They both associate with specific membrane microdomains that are important for the induction of membrane invaginations.^108^ It has been suggested that flotillin-1 is involved in sequestration of different cargoes, such as the GPI-anchored protein CD59, cholera toxin B subunit (CTxB), cationic molecules, proteoglycans and the Niemann–Pick C1-like 1 protein.^109,110^ Depending on the cargo, flotillin-assisted endocytosis can be dynamin-dependent (*e.g.*, epidermal growth factor) or dynamin-independent (*e.g.*, CTxB).^108^ Flotillins regulate several membrane trafficking events, although there is still no evidence that these proteins are essential elements of a specific endocytic pathway. With this in mind, Meister *et al.* proposed the term “flotillin-assisted endocytosis” and assumes an endocytic process that is facilitated by flotillins.^110^

(v) Fast-endophilin-mediated endocytosis (FEME) was recently discovered and is a non-constitutive process that is triggered upon activation of specific receptors including β_1_ adrenergic receptor, EGFR and interleukin-2 receptor.^100^ FEME occurs in the leading edges of the cell membrane where endophilin is recruited by lamellipodin.^111^ This pathway only takes place if pre-enriched endophilin (endophilin-Ptdlns(4,5)P_2_-lamellipodin) is already available at the plasma membrane to bind to the activated-receptor.^112^ FEME is characterized by the rapid formation, usually seconds, of tubulo-vesicular structures of <1 μm at the cell membrane.^100^ To date, the uptake of different NPs through clathrin/caveolae-independent endocytosis has been reported. SPIONs and silica-coated iron oxide NPs (Fe_3_O_4_@SiO_2_) with negative surface charge and a primary diameter of around 17 to 30 nm were shown to be internalized *via* CDC42 (CLIC–GEEC pathway) and caveolae in HeLa cells.^113^ Similarly, Arf6-associated endocytosis together with caveolae-dependent endocytosis and macropinocytosis were associated with the uptake of ∼130 nm polydopamine-coated mesoporous silica NPs (polydopamine@MSNs).^114^ In addition it has been demonstrated that flotillin-1 and -2 are involved in the uptake of 30 nm amorphous SiO_2_ NPs.^115^ The involvement of all the mechanisms in NP internalization is still unclear. There are three possible reasons: (i) clathrin/caveolae-independent endocytosis is not the principal mechanism for NP uptake; (ii) difficulties in distinguishing from other mechanisms, such as clathrin- and caveolae-dependent endocytosis; (iii) lack of knowledge about the process.

### 2.6 Passive uptake

The different entry routes discussed so far are known to be the principal mechanisms for NP uptake. Other processes such as passive diffusion across the outer cell membrane by van der Waals forces or steric interactions (subsumed as adhesive interactions) and pore formation may also be involved as well.^116^ For the study of passive uptake, red blood cells (RBCs) are usually used as models, once they are deprived of most cellular organelles and endocytosis machinery.^117,118^ Quantum dots (QDs) coated with the zwitterionic thiol ligand d-penicillamine (DPA-QDs) of 4 nm radius entered in RBC *via* membrane penetration without pore formation.^119^ Similarly, passive uptake was observed in lung cells after exposure of titanium dioxide (TiO_2_) NPs of 22 nm to rats *via* inhalation.^120^ The same findings were observed when different NP types were exposed to human RBCs.^117^ It was demonstrated that surface charge and material of the particles did not influence their uptake and that internalized particles were not membrane-bound. Also, several positively charged (cationic) NPs have been shown to lead to membrane disruption and formation of nanoscale holes.^121^ This finding is mostly related to experiments where serum-free cell culture medium was used. As an example, cationic AuNPs with high surface charge densities were able to create hydrophilic pores and diffuse into the plasma membrane.^122^

## 3. Influence of NP physicochemical properties on endocytosis

Several factors can affect the internalization of NPs, including NP properties such as composition, size, shape, stiffness, and surface chemistry. These properties are, first and foremost, important for NP stability in the biological environment and, secondly, they can influence the cell–NP interactions and subsequent uptake. The principal parameters to take into consideration for NP stability are complex and have not been considered as a part of the review. For detailed information on this topic several published articles are available.^18,31,123,124^ Herein, the focus is in the influence of different NP physicochemical properties on endocytic mechanisms (Fig. 2).

### 3.1 Size

In general, the size of a substance/particle is considered as one of the most important parameters in endocytosis. Large particles (>500 nm) are known to be internalized only *via* phagocytosis and/or macropinocytosis, while the other endocytic mechanisms are limited in terms of cargo size (maximum size of 200–300 nm).^61^ Several other uptake mechanisms can also be involved in the internalization of NPs. We assume that NP size is not a critical parameter influencing phagocytosis and macropinocytosis mechanisms.^61^ Firstly, because phagocytosis is mostly dependent on protein opsonization and secondly, because macropinocytosis is a non-specific cargo uptake mechanism.^11^*Via* macropinocytosis, cells can engulf NPs of various sizes at the same time and it usually occurs in combination with other mechanisms.^61,73^ In contrast, clathrin- and caveolae-mediated endocytosis seem to be dependent on NPs size.^125,126^ Caveolae-based vesicles are usually smaller (average size ∼60 nm) in comparison with clathrin-based vesicles (average size ∼120 nm), so it is expected that larger NPs preferentially are taken up by the cells *via* clathrin. These vesicle sizes are an average among different cell types, although it is possible to find larger or smaller vesicles in specific cells. Ho *et al.* revealed that 20 and 40 nm PS NPs are more dependent on caveolae-mediated endocytosis than 100 nm PS NPs, as seen in HUVEC cells.^125^ Similar observations were reported in hepatocytes (HepG2) where the uptake of 20 nm AuNPs showed a higher dependence of caveolae in comparison to 40 nm AuNPs.^126^ In addition, studies on BLMVEC revealed that a single caveolae vesicle was able to engulf up to three 20 nm or two 40 nm albumin-coated polymeric NPs.^94^

An important size-related parameter that can affect NP internalization is the aggregation state, once NPs in an aggregated form or as individual particles interact differently with cells.^124^ Opsonization is another factor that can promote NP aggregation, change the surface properties and contributes to NP phagocytosis.^62,127^ The NP aggregation contributes to an overall increase in NP size and may affect their uptake^96^ and intracellular distribution.^128^ For example, Halamoda-Kenzaoui *et al.* showed that well-dispersed SiO_2_ NPs were internalized principally through caveolae-mediated endocytosis, but an increase in the NP agglomeration state shifted to a combination of endocytosis pathways with a predominant role of macropinocytosis.^129^

Size is not only related to the route of internalization but also affects the uptake rate. It has been reported that the internalization of SiO_2_ NPs in A549 lung epithelial cells becomes slower with increasing particle size^130^ and this might be related to the concept of dosimetry as size can determine the behaviour in cell culture medium and thus the delivered dose.^131^ The same observation was reported by Rejman *et al.* where the uptake of fluorescent carboxylate nano-/micro-spheres (50, 100, 200, 500, and 1000 nm), by melanoma cells, revealed to be size-dependent.^132^ A decreased internalization was related to increased microsphere size. In contrast, an increase in the uptake of larger NPs was observed in a different study where AuNPs (13 and 45 nm) were exposed to human dermal fibroblasts.^133^ They showed that 45 nm AuNPs enter the cell more efficiently than 13 nm AuNPs. Yet Li *et al.* demonstrated the interplay between different-sized SiO_2_ NPs (50, 100 and 150 nm) on their uptake in HeLa cells in a co-exposure scenario. When NPs were administered simultaneously to the cells, a competition between different-sized NPs in their cellular uptake was observed. Interestingly, the bigger NPs stimulated the uptake of smaller ones and *vice versa.*^134^ It is important to note that the comparisons between different studies cannot be made without considering the differences between NP types, cells, and experimental conditions. In order to investigate the size effect on NP uptake, the reductionist approach is proposed to minimize all influencing factors except the particle size.

### 3.2 Shape

Shape is a physical property that can also influence the uptake of NPs (for a review please refer to a previous publication).^135^ Xie *et al.* synthesized different gold nanostars, nanorods, and nanotriangles coated with methylpolyethylene glycol and exposed them to macrophage (RAW264.7).^136^ They showed that all AuNPs enter cells through clathrin-mediated uptake. Rods can also be internalized *via* caveolae/lipid raft-mediated endocytosis and triangles *via* additional actin and dynamin-dependent pathways (possibly phagocytosis and/or macropinocytosis). Shape can also have an impact on the increased or decreased uptake of NPs. Vàcha *et al.* described a simulation approach for investigation of the passive uptake of differently shaped NPs.^137^ They suggested that the sphero-cylinders are more efficiently endocytosed compared to spheres of the same diameter. Even though both shapes have the same kinetic barrier for uptake across a membrane, the sphero-cylinders possess the larger volume. For an idealized membrane, it is thermodynamically more convenient to encapsulate a sphero-cylinder than a sphere of the same radius, due to the smaller curvature of the cylindrical part. It has been shown that endocytosis was suppressed for particles with sharp edges (regions with high curvature).^137^ In support of this study, Huang *et al.* evaluated the cellular uptake of mesoporous silica NPs of similar diameter but different aspect ratios (AR): sphere-shaped (AR of ∼1), short rod-shaped (AR of ∼2) and a long rod-shaped (AR of ∼4) in human melanoma cells (A375). The uptake rate of long-rod particles was faster compared to those of short-rod and spherical particles. The most likely explanation for such behaviour could be the larger contact area of rod-shaped NPs with the cell membrane.^138^ Chithrani *et al.* investigated the uptake of differently shaped AuNPs in HeLa cells and found a lower amount of rod-shaped AuNPs within the cells compared to spherical ones.^139^ There are many speculations for this outcome, such as (i) differences in the membrane curvature, (ii) reduction of the available receptor binding sites, (iii) surfactant molecules, which prevent serum proteins from binding onto the NPs surface efficiently, and (iv) non-homogenous protein coating and thus lack of multivalent binding to the receptors.^139^

The interaction between different NPs and cells depends not only on the shape of the NPs, but also on the cell type. Kinnear *et al.* synthesized different gold rods of varied aspect ratios and studied their interaction with macrophages and epithelial cells.^140^ Rods of ∼10–90 nm in length and with small aspect ratios (<5), revealed similar uptake efficiencies in both cells. In contrast, nanorods with higher aspect ratio (>5) were preferentially internalized by epithelial cells, whereas the uptake in macrophages revealed to be shape independent.

### 3.3 Surface charge

The surface charge of NPs influences their behaviour in biological environments due to the presence of biomolecules with various charges.^17^ Several factors can affect the surface charge of NPs, including adsorbed biomolecules, and pH. Herein, besides the design of cationic (positively charged) or anionic (negatively charged) NPs, it is crucial to understand how the NP surface charge changes in such complex environments. Based on the studies published so far, the internalization of cationic NPs is more efficient in comparison to neutral and anionic NPs.^141–144^ 9 nm SPIONs functionalized with different polymers: polyvinyl alcohol (PVA), carboxylate-functionalized PVA (negative charge), thiol-functionalized PVA (neutral charge), and amino-functionalized PVA (positive charge) (amino-PVA) were synthesized, and a higher uptake for amino-PVA-SPIONs in melanoma cells in comparison with carboxy-PVA-SPIONs and thiol-PVA-SPIONs was shown.^145^ He *et al.* investigated the effects of surface charge on cellular uptake of polymeric particles with various sizes (150–500 nm) and zeta potentials between −40 mV and +35 mV in murine macrophages.^146^ They demonstrated more efficient uptake of large particles with higher positive surface charges. Uptake of negatively and positively charged NPs was cell-type-dependent. One of the explanations for the toxicity is that once cationic NPs reach the lysosomes they can lead to their swelling and subsequent membrane rupture.^18^ In contrast, Lunov *et al.* have shown uptake of anionic PS particles by monocyte-derived macrophages (MDMs) mainly *via* clathrin- and dynamin-dependent endocytosis, whereas the cationic ones were taken up *via* macropinocytosis. THP-1 cells did not differentiate between the particle charge and internalized both by macropinocytosis, and clathrin- and dynamin-dependent endocytosis.^147^ The uptake mechanism of charged particles thus strongly depends on the cell- and particle-types.

### 3.4 Surface functionalization

The functionalization of NPs with specific moieties to target specific cellular receptors has been increasing over the years especially as a concept for targeted drug delivery. These receptors usually relate to certain endocytic mechanisms and can be specific for certain cell types or being upregulated in cancer cells.^148,149^ This includes, for example, NP functionalization with EGF^150^ and transferrin^151^ known to be internalized *via* clathrin-mediated endocytosis, but also with albumin^90^ that is associated with caveolae-mediated endocytosis. Besides cell targeting, NPs are functionalized with other molecules in order to increase NP stability and to prolong circulation time. Brandenberger *et al.* have compared the uptake of two different AuNPs, citrate-coated and polyethylene glycol (PEG)-coated, in A549 cells.^128^ The particles were aerosolized on the lung cell surface with an air–liquid cell exposure system to avoid exposure of the particles in the cell culture medium. Citrate-coated AuNPs were taken up by macropinocytosis and by clathrin- and caveolae-mediated endocytosis, whereas PEG-coated AuNPs were internalized *via* clathrin- and caveolae-mediated endocytosis, but not by macropinocytosis.

### 3.5 Topography

Surface nanoscale topography of NPs has recently received particular attention, being considered an important surface property that influences cell–NP interaction.^152^ NPs with different surface topography have been synthesized, including smooth,^153^ rough,^154^ rambutan,^154^ raspberry,^155^ flower,^156^ and virus-like^157^ surfaces. Wang *et al.* investigated the internalization of three different SiO_2_ NPs: solid, mesoporous, and virus-like (spiky tubular rough surface) in HeLa cells.^158^ They showed that virus-like mesoporous SiO_2_ NPs had the fastest internalization rate among the three SiO_2_ NP types. Regarding the entry mechanisms, virus-like mesoporous SiO_2_ NPs were revealed to be internalized mainly *via* caveolae-mediated endocytosis and macropinocytosis, while clathrin-mediated endocytosis was the principal route for the other two NPs.

## 4. Methods to study endocytosis

Our current understanding of cell–NP interactions is primarily due to recent developments in imaging and biochemical analysis. The answers to questions like “Are NPs inside the cell?” and “How many NPs are inside the cell?” are important, but even more important is the answer to “How are NPs internalized?”. Understanding NPs endocytosis mechanisms and its regulation allow the optimization of NP uptake by designing specific NPs and reduction of possible toxic effects. For the research community, *in vitro* and *in vivo* studies of the different endocytic processes require simple and reliable methods, whereas the research focus in this area is on the *in vitro* approach at the single cell level. Different approaches have been used, including chemical and pharmacological inhibitors, genetic approaches, protein and gene expression levels, specific biomarkers, and different microscopy techniques (fluorescence, electron, and atomic force). An overview of these methods will be given, and the pros and cons will be discussed in this section.

### 4.1 Classical chemical and pharmacological inhibitors

The first possibility to evaluate endocytosis of NPs is the use of inhibitors to block the process. The use of classical chemical and pharmacological inhibitors is the most common approach to study NP uptake by cells. These inhibitors are the preferred choice due to: (i) fast action in blocking the uptake route; (ii) inhibition covers the overall cell population; (iii) efficient and cost-effective process.^159^ Nevertheless, when blocking a cellular process, there is always a possibility of side effects (*e.g.*, cytotoxicity) and upregulation of compensatory uptake mechanisms. The major limitations are their lack of specificity and their efficacy variation between cell types, which may require dose-titration studies and the use of different inhibitors.^160^

Several inhibitors have been introduced over recent years, including the recently discovered pharmacological inhibitors itstop 2 and dyngo compounds that inhibit CME and dynamin-dependent endocytosis, respectively (Table 1). Depending on the target pathway, inhibitors can be grouped into: (i) CME; (ii) lipid rafts and caveolae-mediated endocytosis; (iii) macropinocytosis and phagocytosis; (iv) dynamin-dependent endocytosis inhibitors. Compounds that block specifically each type of endocytic mechanism only are not yet available. In this regard, it is suggested to use the combination of different inhibitors in order to avoid ambiguous conclusions. In addition the use of appropriate controls to confirm blocking of a certain mechanism is highly recommended to evaluate the optimal and non-toxic time frame for cell experiments.^161^ The controls include molecules that are known to be internalized *via* the mechanism in study. Transferrin^80^ and lactosylceramide^162^ can be used as controls for CME and caveolae-mediated endocytosis, respectively, whereas dextran 70 kDa and lucifer yellow are controls for macropinocytosis.^163,164^ Several studies in the literature reported the use of other molecules such as cholera toxin B as a control for caveolae-mediated endocytosis. The issue with using cholera toxin B lies in its non-specificity, since this molecule can be internalized *via* different pathways.^165^ Included in CME inhibitors are the classical chemicals such as hypertonic sucrose and potassium depletion (Table 1). Singh *et al.* investigated the effect of both inhibitors on the uptake of cerium oxide NPs.^166^ They revealed that the NP uptake was inhibited in both approaches, which suggested internalization through CME. Even though hypertonic sucrose and potassium depletion have been related to CME, it was also proved that both interfere with other endocytic mechanisms.^167,168^ In a recent study, pitstop 2 was also included to evaluate the internalization of QDs conjugated with single-stranded oligonucleotide-based aptamers (APTs) in A549 cells.^169^ The target of pitstop 2 is CME, and they showed that in the presence of the chemical, APT-QDs were not able to enter into the cell, confirming CME as the predominant mechanism. Even if the mode of action of pitstop 2 is more selective, its non-specificity was also demonstrated, once it was able to block endocytic mechanisms independent of clathrin.^170,171^ Non-specificity is not the only issue with the use of inhibitors, given that they can also affect cellular processes, and cause unusual structural changes and cytotoxicity. Kuhn *et al.* tested four different inhibitors: chlorpromazine and monodansylcadaverine (both CME inhibitors), methyl-β-cyclodextrin (a caveolin-mediated endocytosis inhibitor), and cytochalasin D (a phagocytosis/macropinocytosis inhibitor), in the uptake of PS NPs in A549 and J774A.1 cells.^60^ Cellular damage was observed for A549 cells in the presence of monodansylcadaverine and cytochalasin D, and for J774A.1 cells in the presence of chlorpromazine. Vercauteren *et al.* also verified the cytotoxic effects of chlorpromazine and methyl-β-cyclodextrin in different cell types.^172^ They concluded that the cytotoxic effects caused by the inhibitors were cell-and concentration-dependent.

**Table 1:** Common chemical and pharmaceutical endocytosis inhibitors

Endocytosis inhibitors	Pathway(s) involved	Mechanism	Limitation	Conc. range^a^	Ref.
Hypertonic sucrose	CME	Traps clathrin in microcages	Interferes with macropinocytosis	0.4–0.5 M	167

Potassium depletion		Removes clathrin lattices that associate with cell membrane	Affects actin cytoskeleton	n/a	167 and 173

Cytosol acidification		Inhibits the scission of CCPs from the cell membrane	Affects actin cytoskeleton and interferes with macropinocytosis	10–30 mM NH_4_Cl	174 and 175

CPZ		AP2 inhibitor; blocks endosome recycling	Affects biogenesis of large intracellular vesicles such as phagosomes and macropinosomes	50–100 μM	176

MDC		Stabilizes CCVs	Global changes in the dynamics of the actin cytoskeleton	100–300 μM	177 and 178

Phenylarsine oxide		Inhibits protein tyrosine phosphatases; exact mechanism remains unknown	Inhibits phagocytosis and macropinocytosis	1–20 μM	178

Chloroquine		Reduces the expression of phosphatidylinositol binding clathrin assembly protein (PICALM)	May only affect phagocytic cells and interfere with other endocytic mechanisms	20–400 μM	179

Monensin		Dissipation of a proton gradient	Interferes with endocytic trafficking such as receptor recycling	5–40 μM	180 and 181

ES9-17		Inhibitor of the CHC function; cytosol acidification	Still unknown (discovered recently)	30 μM	182

Pitstop 2		Interferes with binding of proteins to the N-terminal domain of clathrin	Affects most endocytic pathways	10–100 μM	170 and 174

Methyl-β-cyclodextrin	Caveolae-mediated endocytosis/lipid rafts	Cholesterol depletion from the cell membrane	Interferes with macropinocytosis and CME	5–10 mM	183

Filipin		Interacts with cholesterol at the cell membrane	Inhibits CME; unstable and toxic at high concentrations	1–5 μg mL^−1^	184

Statins		Blocks cholesterol synthesis	Affects most endocytic mechanisms	10–100 μM	185

Genistein		Inhibits several tyrosine kinases	Interferes with dynamin and may affect other endocytic processes	200–400 μM	81, 172 and 186

CytD and lantruculins	Phagocytosis and macropinocytosis	Block actin polymerization	Affect most endocytic mechanisms	1–10 μM	60 and 187

Amiloride or its derivatives (EIPA and HOE-694)		Na^+^/H^+^ exchanger pump inhibitor; blocks Rac1 and Cdc42 signaling	Inhibit CME	1 mM for amiloride and 50–100 μM for its derivatives	188 and 189

Imipramine		Inhibits plasma membrane ruffle formation	Still unknown (the mechanism of inhibition is still not fully understood)	5 μM	190 and 191

Wortmannin and LY294002		Inhibit the activity of phosphatidylinositol 3-kinase	Affect most endocytic mechanisms	10 nM–10 μM for Wortmannin and 20 μg mL^−1^ for LY294002	164, 192 and 193

Rottlerin		Inhibits the activity of protein kinase C delta	Affects most endocytic mechanisms	1–3 μM	194 and 195
Dynasore, dynole and dyngo compounds	Dynamin-dependent endocytosis	Inhibit the GTPase activity of dynamin1 and 2	May interfere with cholesterol homeostasis and actin	1–500 μM	196 and 197

a The concentration of inhibitor is cell- and time-dependent.

In short, the choice of inhibitors is cell- and experiment-dependent and once applied they should not cause significant side effects. As most of the inhibitors simultaneously affect different endocytic mechanisms, some caution should be taken when interpreting results and drawing conclusions.

### 4.2 Genetic approaches

In order to overcome the non-specificity of chemical inhibitors, genetic approaches were implemented to change the expression of specific proteins. Included in the genetic approaches are the use of interference RNA (RNAi) to silence specific genes, the use of knockout models, targeted genome editing and mutant proteins.^198^ RNAi refers to small noncoding RNAs (around 20 to 30 nucleotides), which includes micro RNA (miRNA), short interfering RNA (siRNA), short hairpin RNA (shRNA) and piwi interacting RNA (piRNA). Their principal role is the control of gene expression.^199^ siRNA are exogenous synthetic double stranded RNA, and have been used to interfere with specific genes related to the endocytosis process.^160^ The silencing mechanisms of siRNA occurs *via* degradation of the target mRNA, which ends up with the knockdown of the respective protein.^200^ Guggenheim *et al.* investigated the uptake of SPIONs in an epithelial breast cancer cell line (MDA-MB-231), A549, HeLa and THP-1 macrophage-like cells.^201^ They transfected the cells with different siRNA constructs: α-adaptin (AP2 inhibition) siRNA to block CME; caveolin-1 siRNA to block caveolae-mediated endocytosis; PAK-1 siRNA to block macropinocytosis. The uptake of SPIONs was inhibited in different ways by the various siRNA, revealing the involvement of different endocytic mechanisms, which confirms the findings with pharmaceutical inhibitors as described in the previous section.

**Fig. 3:**
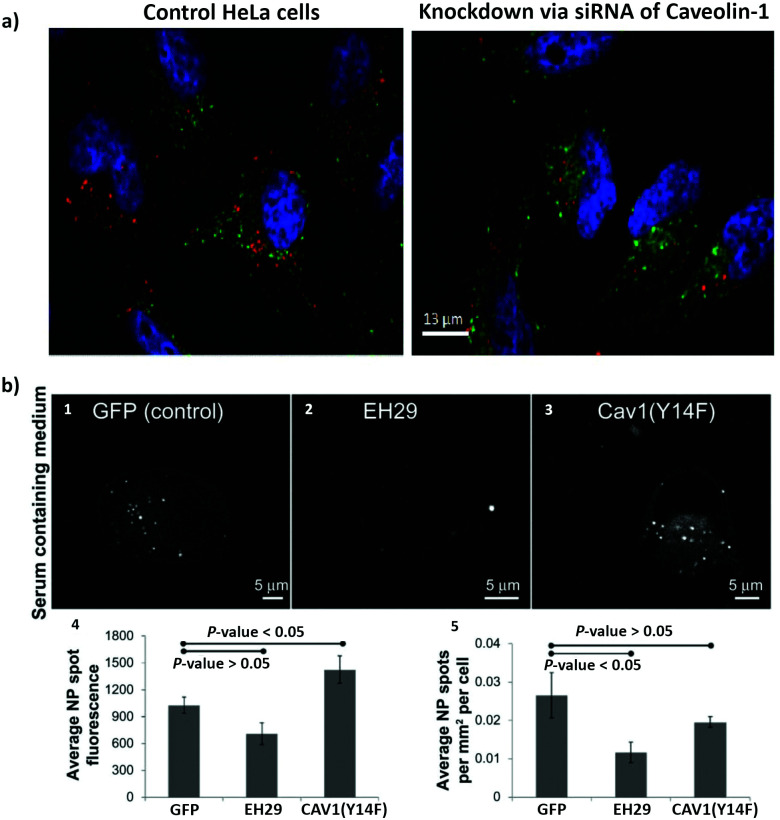
The use of siRNA and mutant proteins to investigate nanoparticle endocytosis. (a) Micrographs of HeLa cells, which were incubated with silica-coated iron oxide nanoparticles (Fe_3_O_4_@SiO_2_) for 4 h; on the left image control cells without siRNA treatment and on the right cells with knockdown of caveolin-1; blue = DAPI (nuclei), green = Transferrin Alexa Fluor® 488 conjugate (cytosol), red = Alexa Fluor® 555 (Fe_3_O_4_@SiO_2_). (b) Representative images of a HeLa cell expressing green fluorescent protein (1), EH29 (mutant form of eps15) (2), and Y14F (mutant form of caveolin 1) (3) incubated with carboxylate-modified polystyrene nanoparticles in serum-containing media. Average fluorescence based on nanoparticle spot (4) and average nanoparticle spot density per cell area (5) for HeLa cells expressing green fluorescent protein, EH29, and Y14F incubated with nanoparticles in serum-containing media. Adapted with permission from (a) ref. 113 and (b)ref. 213. Copyright (a) 2016 Bohmer *et al.*, and (b) 2012 Smith *et al.*, licensed by Dove Medical Press Limited.

Knockout animal models can also be used to investigate the impact of specific proteins in a determined process. Sago *et al.* investigated the *in vivo* delivery of lipid NPs in a caveolin-1 knockout mouse model.^202^ They revealed that NP bio-distribution was affected by caveolin-1 in a cell type-specific manner. An additional genetic approach that is scarcely used in the study of NPs endocytosis, and that might be considered in future studies, is the use of clustered regularly interspaced short palindromic repeats (CRISPR)/CRISPR-associated (Cas) systems.^203^ The CRISPR–Cas system is part of the RNA-mediated adaptive defense of prokaryotes and acts by cleaving the nucleic acids of invading viruses.^204^ These systems have been used to modify, regulate, or label specific genes in several cells and organisms.^205^ CRISPR-Cas9 is a novel efficient method for gene knockout and involves the Cas9 protein, an enzyme responsible for DNA cutting and a single guided RNA (sgRNA), which guides Cas9 to a specific location in the DNA sequence.^204,205^ This approach has been used to prepare AP2M1,^206^ CAV1,^207^ dynamin^208^ and Rac^209^ knockout cells. Patel *et al.*, studied the intracellular delivery of mRNA from lipid NPs.^210^ They introduced CRISPR-based genetic perturbations on the lysosomal pathway of haploid cells (HAP1) *via* knockout of Rab5A, Rab4A, or Rab7A. These proteins can be found on early, recycling, and late endosomes, respectively. They concluded that late endosome/lysosome formation is essential for functional delivery of exogenously presented mRNA. The methods described above involve the inhibition of the expression of a protein of interest. There is still the possibility of using mutant proteins, where mutations (*e.g.*, deletions) are introduced into the gene (*i.e.*, DNA sequence) leading to the expression of dysfunctional proteins.^211^ Dominant negative mutations are the most common mutations to investigate the involvement of specific proteins in endocytosis. The overexpression of these mutated proteins in comparison to the endogenous wild-type proteins may culminate with non-functional proteins, inactive or hyperactive proteins [*e.g.*, GTPases (Rho and Rab) and kinases (PAK and PKC)].^198^ A mutant form of dynamin, K44A, was one of the first mutated proteins used to study the importance of dynamin in the endocytosis of transferrin *via* CME.^212^ It was shown that internalization of transferrin in mammalian cells is blocked in the presence of dysfunctional dynamin. In a different study, Smith *et al.* investigated the effect of two mutant proteins, eps15 (EH29) and caveolin1 (Y14F), on the uptake of 20 nm carboxylate-modified PS NPs in HeLa cells (Fig. 3).^213^ They observed that the expression of the inhibitor of clathrin-mediated endocytosis eps15 (EH29), but not the dominant negative caveolin-1 (Y14F), significantly reduced NP uptake.

The genetic approaches discussed in this section present the advantage to be more specific than the chemical inhibitors, but they also have some limitations. Inhibition of protein expression may lead to compensatory mechanisms in the cell.^160^ Depending on the required time to silence a gene the cell may adapt and change gene expression.^198^ When transfecting cells with mutant proteins it is important to know if those proteins can be involved in more than one endocytic pathway.^104^ In addition, to get a dominant effect of the mutant protein, a certain level of overexpression (in comparison with the endogenous wild-type) should be achieved. Therefore, combining different approaches and the inclusion of appropriate positive and negative controls are crucial to avoid misinterpretations.

### 4.3 Protein and gene expression levels

**Fig. 4:**
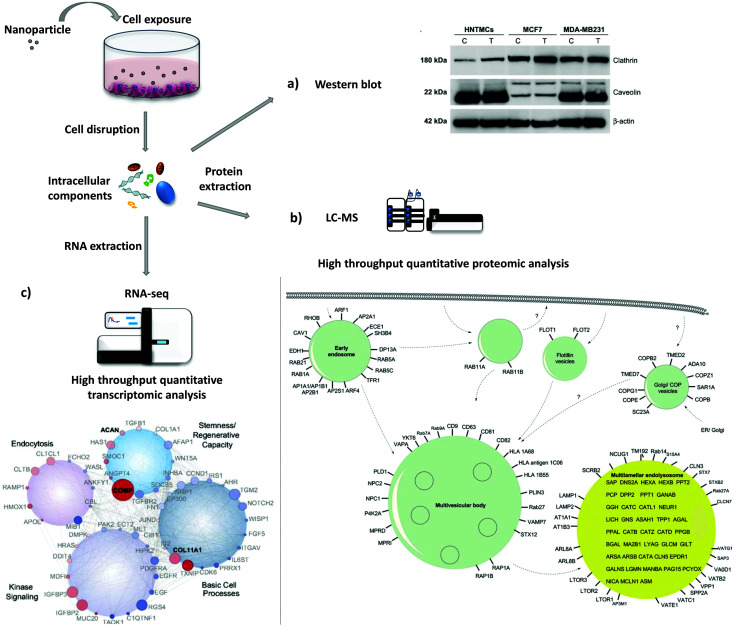
Evaluation of protein and gene expression upon nanoparticle exposure. Following nanoparticle exposure, cells can be disrupted and protein and RNA can be extracted. (a)–(c) are examples of possible downstream applications. (a) Western blot analysis of expression levels of clathrin and caveolin in MDA-MB231, MCF7, and human nontumor mesenchymal cells (HNTMCs). Control (C) and treated (T) cells with maghemite–rhodium citrate (MRC) nanoparticles (NPs) for 6 h. (b) Schematic illustration of intracellular nanoparticle trafficking based on gene ontology (GO) terms vesicle and lysosome. HeLa cells were exposed to superparamagnetic iron oxide polystyrene nanoparticles for 20 h and vesicles were magnetically separated. Label-free quantitative liquid chromatography-mass spectrometry (LC-MS) was performed followed by bioinformatic data analysis (DAVID ontology analysis). Only the proteins magnetically enriched by a factor of >2-fold are shown. (c) Analysis of the whole-transcriptome level by high-throughput sequencing (RNA-seq). Human mesenchymal stem cells (hMSCs) were exposed to nanosilicates for 7 days. At the bottom, scheme of the gene network, which comprises genes with different degrees of expression and high statistical significance (red, up-regulated; blue, down-regulated; size increases with significance). Reproduced with permission from (a)ref. 221 and (c)ref. 222. Adapted with permission from (b)ref. 220. Copyright (a) 2017 Chaves *et al.*, licensed by Dove Medical Press Limited, (b) 2016 American Chemical Society and (c) 2018 Carrow *et al.*, licensed by Creative Commons Attribution-NonCommercial-NoDerivatives License 4.0 (CC BY-NC-ND).

The study of protein and gene expression levels, including proteomic and transcriptomic analysis enables the identification of the different proteins and transcripts associated with the process of endocytosis (Fig. 4). For example, it is possible to isolate endocytic vesicles and investigate the associated proteins by mass spectrometry (MS)-based proteomics.^214^ This approach, besides allowing the identification of proteins involved in endocytic uptake processes, also offers the possibility to quantify the protein levels. The two most widely used methods for high-throughput quantitative proteomic analysis in complex samples are gas chromatography MS (GC-MS) and liquid chromatography MS (LC-MS).^215^ LC-MS has been used as a bottom-up proteomics approach to analyze clathrin-coated vesicles,^216,217^ lipid rafts^218^ and phagosomes.^219^ Hofmann *et al.* included proteomic analysis, based on LC-MS, to investigate the uptake of superparamagnetic iron oxide PS NPs in HeLa cells (Fig. 4b).^220^ The identified proteins, including Arf1, together with other approaches, *i.e.* colocalization studies and the use of inhibitors, suggested the involvement of macropinocytosis in the internalization of NPs. Instead of using high-throughput analysis, there is the possibility to look directly at a few specific proteins. As a simple and more direct approach, protein levels can be determined *via* immunoassays [*e.g.*, enzyme-linked immunosorbent assay (ELISA) and western blot]. Chaves *et al.* studied the expression levels of two proteins, clathrin heavy chain and caveolin 1, upon exposure to maghemite–rhodium citrate NPs in different cell types (breast cancer cell lines, MCF7 and MDA-MB-231, and primary human non-tumor mesenchymal cells) (Fig. 4a).^221^ For all cells, an increase in the clathrin heavy chain protein levels but not in caveolin-1 was observed, suggesting CME as the principal mechanism for internalization of maghemite–rhodium citrate NPs.

Instead of looking directly at protein levels, it is possible to evaluate the expression of particular genes (transcripts). This approach can be used to complement protein analysis. Here, the expression of certain genes or the analysis of the whole transcriptome (*e.g.*, DNA microarrays and RNA-seq) can be performed. It is important to consider that transcript (mRNA) levels may not correlate with protein levels due to post-transcriptional modifications. Carrow *et al.* investigated the changes in the transcriptome profile of human mesenchymal stem cells induced by two-dimensional nanosilicates (Fig. 4c).^222^ They revealed significant changes in the expression level of 4.068 genes, where a large part has been found to be involved with endocytosis. In addition, genes related with CME were affected as well, revealing the involvement of CME on the uptake of nanosilicates.

Transcriptomic and proteomic analyses are powerful techniques that provide a large amount of information regarding endocytic processes and other biological processes (*e.g.*, cell proliferation, inflammation, and apoptosis).^223^ These are expensive, time-consuming and laborious approaches, requiring fundamental support of bioinformatics and biostatistics.^224^ Investigation of a small group of transcripts and/or proteins are simpler and faster; however some proteins may be connected with different endocytic mechanisms and their expression may not change considerably.^215^ A crucial aspect to consider when looking for transcript and protein levels is the selection of the most appropriate time points. Depending on the experiment (*i.e.*, cell type, NPs properties, administered dose, *etc.*) the expression of proteins involved in endocytosis can change. Therefore, it is recommended that a time-course analysis is carried out, involving different time points, to be able to see possible significant effects.

### 4.4 Endocytic markers and microscopy analysis

**Fig. 5:**
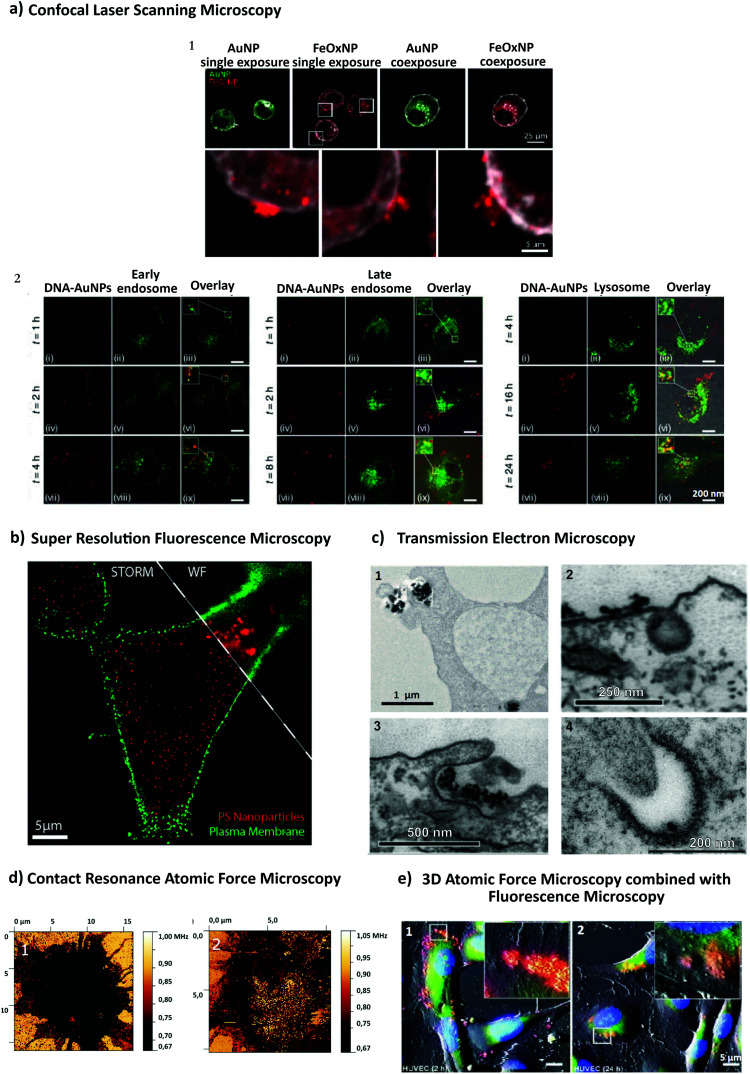
Different approaches to study NP endocytosis. (a) Confocal laser scanning microscopy reveals the effect of AuNPs and FeO_*x*_ NPs uptake after monodansylcadaverine treatment of macrophage cells; the uptake of FeO_*x*_ NPs is partially blocked in the presence of the inhibitor as shown by the aggregation of NPs in the outer side of cell membrane (1). Fluorescence microscopy images showing co-localization of DNA-decorated AuNPs (fPlas-gold) (red) with early endosomes (green), late endosomes (green) and lysosomes (green) over time (2). (b) Super-resolution fluorescence microscopy, stochastic optical reconstruction microscopy (STORM), of the uptake of 80 nm polystyrene NPs (red) in HeLa cervical carcinoma cells (plasma membrane, green) in comparison with conventional wide-field microscopic techniques. (c) Transmission electron microscopy (TEM) images of silica NPs being internalized *via* phagocytosis (1). TEM micrographs of clathrin-mediated endocytosis (CME) (2) and macropinocytosis (3) of magnetic cobalt ferrite NPs coated with polyacrylic acid (PAA). Electron microscopy image shows two nanoparticles (22 nm) located within a caveolin of a lung epithelial cell (A549) (4). (d) Contact resonance frequency (CRF) map, based on contact resonance atomic force microscopy (CR-AFM), of an empty microglia cell (1) and of a microglia engulfed with Fe_3_O_4_ NPs (2). (e) Three-dimensional AFM (3D-AFM) combined with fluorescence microscopy; perylene-labeled SiO_2_ NPs were visible on the outside of HUVEC cell membranes after exposure for 2 h (1); after 24 h (2) of incubation time, the cell surface is characterized by homogeneous distribution of only small humps, indicating intracellular localization of applied NPs. Adapted with permission from (a1) ref. 24, (a2) ref. 425, (b) ref. 426, (c1) ref. 427, (c2 and c3) ref. 428, (c4) ref. 429 (d) ref. 430 and (e) ref. 431. Copyright (a1) 2017 Vanhecke *et al.*, (a2) 2017 Liu *et al.*, (b) 2016 American Chemical Society, (c1) 2019 Martin *et al.*, (c2 and c3) 2015 Lojk *et al.*, (c4) 2011 John Wiley and Sons, (d) 2017 Royal Society of Chemistry and (e) 2013 John Wiley and Sons.

Recent advances in imaging, including electron, atomic force and super resolution light microscopy techniques, contribute to the understanding of NP uptake by cells and intracellular trafficking (Fig. 5).^11^ Microscopy comprises a variety of electron and light microscopes to visualize smaller scale structures in a sample by presenting a magnified image.^225^ Importantly, not all microscopes are able to resolve nanoscale structures such as NPs and subcellular compartments (*e.g.*, clathrin and caveolae vesicles). In light microscopy, the resolution is limited due to the diffraction limit of light.^226^ In this sense, new advances in this field, with a focus on fluorescence techniques, improved the resolution by increasing spatial resolution [*e.g.*, confocal, total internal reflection fluorescence (TIRF) and structure illumination microscopy (SIM)] or by bypassing the diffraction limit using super resolution fluorescence microscopy [*e.g.*, stimulated emission depletion (STED), expansion microscopy (ExM) and single molecule localization microscopy (SMLM)].^225^ The importance of digital image restoration should be noted, including de-convolution algorithms that may increase the resolution by 2–3 fold.^227^ Super-resolution approaches were developed based on pre-existent microscope setups, wide-field, TIRF and confocal, to overcome the diffraction limit by taking advantage of particular fluorophore properties.^228^ In order to apply these techniques in biological systems, specific cellular structures or molecules have to be labeled with fluorophores. Fluorophores can be grouped into organic dyes [*e.g.*, FITC], and biological fluorophores [*e.g.*, green fluorescent protein (GFP)].^229^

Endocytic mechanisms can be differentiated based on the involvement of particular molecules called markers, such as clathrin in CME and caveolin-1 in caveolae-mediated endocytosis. Three main approaches are used to label these endocytic markers: (i) direct labeling *via* immunofluorescence, which is limited to fixed samples *i.e.*, dead cells; (ii) cells expressing GFP-fusion proteins, where GFP-transcripts can be integrated (stable transfection) or not (transiently transfection) into the genome;^230^ or (iii) fluorescently labeled proteins, which are taken up by cells through specific mechanisms such as transferrin (clathrin-mediated endocytosis) and are finally located in the targeted vesicle. Nevertheless, besides the labeling of endocytic markers, NPs should also be tagged with fluorophores or some NPs can inherently emit specific optical signals (*e.g.*, QDs and AuNPs).^231,232^ The identification of the internalization process of NPs is usually performed *via* fluorescence co-localization analysis of endocytic biological markers and NPs.^233^ This approach allows determining whether both, *i.e.* marker and NPs, localize within the same sub-cellular structures, such as NP co-localization with clathrin.^234^ In this case, it is possible to determine the extent of spatial overlap of both fluorophores (co-occurrence) and the proportional relation with each other (correlation).^235^ Peñaloza *et al.* studied the internalization mechanism of poly(3-hydroxybutyric acid-*co*-hydroxyvaleric acid) (PHBV) NPs in HeLa and human ovarian adenocarcinoma SKOV-3 cells.^236^ For that, NPs were functionalized with a fluorophore, Nile Red, and detection of caveolin-1 and early endosome antigen 1 (EEA1) was performed *via* immunofluorescence. Co-localization fluorescence microscopy in HeLa cells revealed that after 15 minutes NPs co-localize with caveolin-1 and EEA1, which suggests their cellular entry through caveolae-coated vesicles. In SKOV-3 cells, NPs did not co-localize with caveolin-1, indicating that PHBV NPs are internalized *via* a mechanism independent of caveolae. In addition, instead of NP co-localization with specific cell compartments, some researchers opt for co-localizing them with specific cargoes (*e.g.*, fluorescently labeled cholera toxin B, transferrin and dextran).^237,238^ Dynamics and intracellular trafficking of NPs can also be studied in living cells. Li *et al.* applied a super-resolution photoactivated localization microscopy (PALM) with single particle tracking (SPT), which allows them to study the dynamics of PS NP interaction with cells with high spatial and temporal resolution.^239^ They concluded that NPs were internalized in two different ways *via* CME: (i) predominantly, NPs first attached to the cell membrane and clathrin-coated pits (CCP) formed at that site; (ii) NPs diffused on the membrane and translocated through a preformed CCP. Additionally, they revealed that 20 and 40 nm PS NPs were readily internalized *via* CCPs, whereas large PS particles (200 nm) were taken up by cells through a different mechanism.

Electron microscopy (EM) represents a different technique where a beam of electrons takes the place of light rays. EM is used to access the ultrastructural information of biological and non-biological specimens, providing a resolution in the nanometer range.^240^ In particular, transmission electron microscopy (TEM) provides valuable insight into the localization of NPs in the cellular compartments.^241^ Several drawbacks are associated with TEM, including the laborious sample preparation techniques, which includes fixation, dehydration, embedding, sectioning (<100 nm) and staining,^240–242^ thus does not allow one to visualize dynamic events. TEM is regularly used as a characterization method for NPs and it is also applied to visualize the localization and distribution of NPs within tissues, cells and subcellular structures.^243^ When investigating the cellular uptake mechanisms of NPs it is important to differentiate between cellular compartments. However, merely visualization of morphological appearance (*e.g.* clathrin vesicles, and early and late endosomes) can be challenging.^240^ To overcome these limitations, immunogold labelling of subcellular compartments can be performed.^244^ It is important to consider that organic NPs, such as polymer NPs, are composed by elements with a lower atomic number that are commonly present in the cell (*e.g.*, carbon, hydrogen, nitrogen, and oxygen), which remarkably increases the difficulty to analyze them by EM techniques.^243^ Inorganic NPs such as gold and heavy metal NPs are easily identified inside or outside the cell due to their high electron density that enhances imaging contrast.^240^ Dobay *et al.* investigated the subcellular distribution of AuNPs in lung epithelial cells by TEM.^245^ They showed that AuNP uptake by cells occurs through different mechanisms including caveolae-dependent endocytosis. TEM has been applied in combination with different endocytic inhibitors to analyze the uptake route of polymer-coated AuNPs in A549 cells.^161^ It has been demonstrated that caveolae-mediated endocytosis was the main uptake mechanism for AuNPs.

Atomic force microscopy (AFM) is a powerful imaging tool that enables visualization and manipulation of biological samples, from single molecules to living cells.^246^ It can operate in liquid environments and allows the analysis of biomolecules and cells with resolution comparable to that of EM techniques (sub-nanometer).^247^ An AFM unit is composed of a flexible cantilever where an ultra-sharp tip is mounted. Topographical and mechanical information is obtained based on a piezoelectricity-driven scanning cantilever that measures the force between the sample and the sharp scanning probe tip.^248^ The principal drawbacks of imaging living cells are associated with tip contamination and possible interactions between the tip and the cell, which may lead to remodeling of events.^247^ Even if AFM provides three dimensional (*x*, *y*, *z*) information on tip position, cellular membrane deformations may occur due to the challenge in identifying the exact contact point between the tip and sample.^246^ In order to increase its potential, fluorescence microscopy techniques such as confocal and TIRF can be combined with AFM that allows multi-parameter characterization of single biomolecule structures and dynamic interactions.^246^ Hor *et al.* were able to study CME in human melanoma skin cells (SKMEL) by using AFM combined with fluorescence microscopy.^249^ In order to access the intracellular compartments, the upper membrane of the cells was removed using sonication (unroofing technique). By using genetically modified SKMEL cells that express a turquoise fluorescent protein (Tq2) along the clathrin light chain, they were able to correlate between the fluorescence and the topographical images of the clathrin-coated pit.

## 5. Methods to (semi)quantify NP uptake

Due to high interest in NPs applications for therapeutics delivery, accurate estimation of internalized NPs in cells is crucial. As an example, Dai *et al.* quantified the cancer cell-targeting efficiencies of the anti-cancer drug trastuzumab (Herceptin) and folic acid-coated gold and silica NPs in mouse tumor models.^250^ It has been shown that only 2% of cancer cells associated with the intravenously administrated NPs as assessed by flow cytometry. Less than 0.0014% of intravenously administrated NPs were delivered to targeted cancer cells as shown by inductively coupled plasma-mass spectrometry analysis. The majority of the NPs were either trapped in the extracellular matrix or taken up by perivascular tumor-associated macrophages. For this reason, it is crucial to develop methods to quantitatively assess the number of delivered drug-loaded NPs and to improve therapeutic efficacy. NP detection within single cells is challenging due to their small size.^241^ The development of highly sensitive methods and imaging techniques offers more reliable and reproducible results. There are several direct microscopic and analytical methods^251^ to quantify NP uptake by using fluorescently-labeled NPs,^252^ QDs,^253^ and magnetic NPs.^254^ Fluorescence microscopy may provide spatial information (*e.g.* subcellular localization) on NP uptake,^255^ but it is not optimal to estimate the total number of intracellular NPs. Also NP aggregation affects the estimation as it may cause difficulties to distinguish between intracellular and cell-associated NPs. A fluorescence signal depends on the excitation source, the number of fluorophores per NPs, the quantum yield of fluorophores or the NP, and the detector sensitivity. Therefore, the signals have to be compared with a control sample and are given as relative values, providing semi-quantitative outcomes.^256^

In contrast, indirect methods such as inductively coupled plasma (ICP) techniques including ICP-optical emission spectroscopy (OES) and ICP-mass spectrometry (MS) can measure total mass of the NP elements.^257,258^ Quantification of NPs uptake using ICP-MS looks promising, but it requires sample destruction and does not provide any information about NP distribution within the cell.^259^ It is hard to draw a conclusion on which method is the most suitable for NP quantification, as it strongly depends on NP characteristics and therefore it is recommended to combine different methods, *e.g.* flow cytometry and confocal laser scanning microscopy^260^ or stereological sampling techniques and TEM.^259^ Recently, many new techniques have been described for NP quantification in cells, and only some of them will be described here.

### 5.1 Flow cytometry

Flow cytometry (FC) is a semi-quantitative fluorescence-based method for single-cell analysis,^261^ where a sheath fluid containing cells, loaded with fluorescent NPs, travels in a laminar flow regime. At the interrogation point, cells are sequentially excited by multiple lasers. Photomultiplier detectors capture the fluorescence emission signals. Every single cell is analyzed for visible light scatter to obtain information about the cell size (forward scatter or FSC) and granularity (side scatter or SSC), independently of the fluorescence signal.^261^ Further on, multiple lasers can obtain information about cell viability (according to live/dead staining) as well as integrated fluorescent signals coming from intracellular/associated NPs, such as QDs,^262^ TiO_2_^263^ or fluorescently labeled SiO_2_.^264^ FC is a rapid and sensitive method, providing information about thousands of cells (events) in one sample. Studies show that it is feasible to quantify in flow cytometry after determining the average fluorescence intensity per particle.^264^ However, this technique does not distinguish between intracellular and membrane-associated NPs.^130,263^ In this regard, flow cytometry can be used to complement confocal microscopy to obtain both, semi-quantitative and qualitative, information about NPs–cell interactions.^130,252,265^ The advantage of this technique is the ability to analyze numerous cells and fluorophores simultaneously, as well as to correct the spectral overlap between fluorophores (*i.e.* compensation). The latter is highly important in co-exposure studies. Finally, untreated and unstained control samples need to be included to achieve an optimized analysis of cell–NPs associations and to prevent autofluorescence detection.^241^ Another interesting aspect to consider is the possible NP interference with the fluorescent dyes used in FC. Two case studies addressed SiO_2_ NP and AuNP interference in the FC Annexin V/propidium iodide assay (quantifies apoptotic and necrotic cells). Both NPs caused distinct interference reactions. In the absence of serum proteins, SiO_2_ NPs induced false-positive signals, whereas gold NPs provoked fluorescence quenching effects.^266^

For the design of NPs used in biomedical applications, it is important to obtain quantitative data about NPs uptake and at the same time information about spatial distribution of the NPs in live target cells. For this reason, imaging FC has been developed recently, which enables wide field imaging combined with high-throughput. It has been shown to be an efficient method for the quantification of nanoliposomes uptake in lymphocyte cells.^267^

### 5.2 Fluorescence microscopy combined with digital analysis

Confocal laser scanning microscopy (CLSM) is a commonly used imaging method to assess NPs–cell interactions for various biological systems.^251^ It provides insight into the uptake and intracellular fate of NPs in fixed and living cells,^268,269^ thus reflecting their spatial distribution. Discovery and development of fluorescent dyes offered new possibilities for CLSM application, such as use of NPs–antibody conjugates,^270^ particles loaded with dyes^271^ or NP functionalization.^272^

Resolution limit of CLSM and the small NPs size prevent distinguishing between single particles and NP agglomerates. Alternatively, fluorescent particle events can be counted, where one event represents an individual compartment (*e.g.* endosome or lysosome) loaded with NPs.^241^ Therefore, only relative differences in NPs uptake per cell can be quantified by comparing different concentrations or time points.^273^

### 5.3 Elemental analysis

Elemental analysis techniques are available to quantitatively assess the elemental mass of NPs due to a very sensitive detection range (detection limit is less than 1 μg L^−1^). The most common techniques are ICP-MS, atomic emission spectrometry (AES) and OES.^274^

Several research groups reported ICP-MS as a valuable technique to quantify NPs in various biological samples.^139,275–279^ For example, Sadauskas *et al.* quantified the gold content in mice liver and lungs by ICP-MS.^278^ ICP-MS/AES/OES has been used to investigate mouse brain samples for the presence of ceric oxide,^277^ uptake of the metal oxide NPs by human aortic endothelial cells (HAECs)^276^ and internalization of SV40-modified AuNPs in HeLa cells.^275^

Elemental analysis techniques have received considerable attention due to their sensitivity and precise quantification of various elements, especially metal- and metalloid-based NPs. Another advantage is the ability to quantify elements intracellularly. Unfortunately, sample digestion prior to analysis is inevitable and thus, distinguishing between internalized and membrane-associated NPs is not possible.^241,251^ In addition, ICP is not an optimal technique for quantification of silica, titania, and polymeric NPs due to intense polyatomic interferences.^280^

In order to obtain high-quality data, ICP techniques can be combined with optical and electron imaging methods such as contrast microscopy,^275^ TEM and CLSM^281^ that also provide spatial information.

### 5.4 Focused ion beam-scanning electron microscopy (FIB-SEM)

When paired with scanning electron microscopy (SEM), focused ion beam (FIB) provides high-resolution 3D images of the NP distribution at the single-cell level. The principle of FIB-SEM is to create an image of the surface layer by using a beam of charged gallium ions (Ga^+^), with a diameter of ∼10 nm. This technique enables both milling (with the FIB) and imaging (with the SEM) a specific region of interest in a fixed sample without moving the stage. By collecting many 2D-image stacks (*i.e.* parallel sections of constant thickness), we can reconstruct a 3D image of a sample.^282^

Recently, FIB-SEM has become popular in nanoscience to visualize 3D nanostructures in a variety of biological specimens.^283,284^ James *et al.* describe FIB-SEM as a promising 3D approach for imaging and quantification of ZnO distribution and dissolution within human macrophages.^285^ FIB-SEM has also been used in combination with fluorescence microscopy and flow cytometry to study uptake and cellular distribution of silver NPs in mesenchymal stem cells.^286^

FIB-SEM automatically generates 3D images and has superior *z*-axis resolution (<10 nm). FIB operates at various angles and acts as a precise blade, thus creating a sample free of artifacts. The weakness of this technique is the time-consuming sample preparation and data collection steps, which can last up to several days.^282^ Furthermore, the process itself is destructive and does not allow re-imaging.^287^ In the future, FIB-SEM could be paired with other imaging techniques, which could open up new areas in nanoscience.^282^

### 5.5 Other methods

In addition to the above-described methods, there have been several other methods developed to assess NP uptake. Developments in environmental electron microscopy provide the possibility to study dynamic NP uptake at the subcellular resolution.^288^ Another approach is to combine stereology (*i.e.*, estimation of three-dimensional structures from two-dimensional sections) and electron microscopy, allowing accurate assessment of the number of internalized NPs per cell.^259^ Brandenberger *et al.* applied this method to analyze the uptake of plain (*i.e.*, citrate-stabilized) and PEG-coated AuNPs in A549 cells.^128^ The results showed the intracellular presence of ∼2500 and ∼3500 plain AuNPs per cell after 1 h and 4 h of exposure, respectively. In contrast, only ∼500 (1 h) and ∼1000 (4 h) PEG-coated AuNPs per cell were found inside the cells. This approach has been shown to be beneficial for estimating the relative distribution of NPs inside specific compartments (*i.e.*, nucleus, mitochondria, endoplasmic reticulum and Golgi) and to follow the NP intracellular trafficking.

Furthermore, surface-enhanced Raman spectroscopy (SERS) provides a detailed image of NPs–cell interactions.^289^ Next, correlative light and electron microscopy (CLEM) has become a powerful tool in studying NPs uptake.^290^ A combination of different approaches should be considered in order to correctly evaluate the results. The limitations of each system need to be considered for the correct interpretation of the results.

## 6. Stimulating NP endocytosis

In order to achieve high treatment efficacy, the enhanced uptake and accumulation of NPs in target cells is crucial. NPs uptake can be stimulated by their functionalization with targeting ligands on the surface or by co-exposure with other NP types. Besides, various bio-inspired molecules have been reported to significantly affect NPs cellular uptake. Table 2 represents the summary of molecules, which can stimulate NPs uptake.

**Table 2:** Summary of main substances related to increased NPs uptake

	Stimulus			Particles				Cell types	Summary of effects	Ref.
	Type	Concentration	Exp. time	Material	Size	Concentration	Exp. time			
**Inflammatory molecules**										
Cytokines	LPS, IFN-γ, IL-10	20–1000 ng mL^−1^	40 h	Silicon oxide	26, 41, 1750 nm	NPs: 50 μg mL^−1^	1–3 h	MDM, AM, TAM, THP-1	IL-10 ↑ uptake of 26 and 41 nm NPs in MDMs and THP-1	22
						Microparticles: 100 per cell				
	IL-10	20 ng mL^−1^	7 days	HDL, LDL	n/a	40–50 μg mL^−1^	4 h	RAW264.7, MDM, Kupffer cells	IL-10 ↑ HDL and LDL uptake in RAW264.7	298
	LPS, IFN-γ, IL-10, IL-4	10–100 ng mL^−1^	24–48 h	PS	30, 50, 100 nm	10^13^–10^15^ per mL	8–24 h	BMDM	LPS and IFN-γ ↑ uptake of 100 nm NPs by M1 macrophages	292

Serum	HS	10%	0.5–6 h	Silica	50, 100, 200, 500, 1000 nm	25 μg mL^−1^	0.5–6 h	THP-1	HS ↑ uptake of NPs by M2 macrophages	295
	BSA	n/a	n/a	MSN	50 nm	50–500 μg mL^−1^	2–4 h	RAW264.7	BSA ↑ uptake of 100 nm NPs by M2 macrophages	296

Immuno-globulins	CD200	30 molecules per μm^2^	n/a	PLGA	7 μm	10 per cell	12 h	BMDM, monocytes	CD200 ↑ phagocytosis of PLGA NPs	355

**Co-exposure with different nano-/micro-particles**										
NPs	SiO_2_	10 μg mL^−1^	0.5 h, 4 h	TiO_2_	<50 nm	10 μg mL^−1^	0.5–4 h	BMDM	SiO_2_ and TiO_2_ NPs synergistically ↑ macrophage inflammatory response	301
	Au	38.6 μg mL^−1^	24 h	FeO_*x*_	28 nm	54.8 μg mL^−1^	24 h	J774A.1	↑ uptake and colocalisation of Au and FeO_*x*_ NPs in co-exposure	24
	Carbon black	0.5 μg mL^−1^	25 h	Fe_2_O_3_	45 nm	0.5 μg mL^−1^	25 h	A549	↑ ROS; intracellular redox reaction between CB and Fe^3+^	23
	Ag	0.3 μg mL^−1^, 3.5 μg mL^−1^	4 h	HgCl_2_, CdCl_2_	n/a	2.8–28 μM HgCl_2_	4 h	HepG2	↑ uptake of HgCl_2_ in co-exposure with Ag NPs	304
						0.15–1.5 μM CdCl_2_				
	SiO_2_	10 μg mL^−1^	24 h	As	n/a	1 μg mL^−1^	24 h	HepG2, HT1080	↑ intracellular level of As in the co-exposure with SiO_2_	303

Micro-particles	PSL	1–10 per cell	2 h, 4 h	R-PLGA	3 μm	1–10 per cell	2–4 h	NR8383	↑ uptake of PSL in the presence of R-PLGA	302

**Bio-inspired molecules**										
Cell-penetrating peptides	TAT	10 μM	n/a	Ag, Au, IONPs, QDs, BSA, dextran	20–50 nm	Ag/AuNPs: 0.27–0.79 nM; IONPs, QDs: 50 μg mL^−1^	1 h	CHO, H1975	TAT-Ag ↑ the uptake of bystander Ag NPs	195
		n/a	n/a	MSN	50 nm	100 μg mL^−1^	2–4 h	RAW264.7	Cys-TAT ↑ uptake of MSN NPs	296
		n/a	4 h	PLGA	189 nm	n/a	4 h	Caco-2	TAT-PLGA conjugation ↑ PLGA uptake	327
	Pep TGN	n/a	n/a	PEG–PLGA	100 nm	50 μg mL^−1^	0.5–2 h	bEnd.3	Conjugation with Pep TGN ↑ NPs cellular uptake	322
	SR9	6 μM	n/a	QDs	n/a	100 nM	1 h	A549	SR9 ↑ QDs uptake	324
	R11	n/a	n/a	SPIONs	38 nm	50 μg mL^−1^	4 h	T24, SV-HUC	↑ uptake of SPIO-R11	325
	Sta-R8	n/a	n/a	FA–PLGA/PK3	137 nm	n/a	2 h	RAW264.7	FA and Sta-R8 ↑ uptake of PLGA NPs	326

Bacteria	*Listeria*	n/a	n/a	n/a	40, 200 nm	10^9^ particles	0.5–72 h	KB	↑ cargo uptake in the presence of bacteria	335

Vitamins	Folic acid	n/a	n/a	MSN	110–168 nm	0.01–20 μg mL^−1^	2 h	Y79	↑ uptake of FA-conjugated MSN *via* receptor-mediated endocytosis	341
		n/a	n/a	ZnO	20 nm	1.25–10 mg mL^−1^	12–48 h	U87MG	↑ uptake of FA-conjugated ZnO *via* receptor-mediated endocytosis	340
		n/a	n/a	Au@SiO_2_	105 nm	n/a	0.5–3 h	MCF-7, MDA-MB-231	↑ uptake of Au@SiO_2_ in MDA-MB-231 *via* receptor-mediated endocytosis	339
		n/a	n/a	Pentablock copolymer	21 nm	n/a	4–24 h	HeLa, HEK293	↑ uptake of FA-conjugated NPs *via* receptor-mediated endocytosis	338
	Vitamin B_12_	n/a	n/a	YG™	50, 100, 200 nm	n/a	O/N	Caco-2	Modification with vitamin B_12_ ↑ YG uptake; highest uptake for 50 nm YG	351
	Vitamin C	300 mg mL^−1^	n/a	ZnO	112–196 nm	15 mg L^−1^	2–24 h	GES-1	Vitamin C ↑ endocytosis of ZnO and ↑ cytotoxicity	352
	Vitamin E	n/a	n/a	PLGA, PS	200, 500, 1000 nm	n/a	2 h	Caco-2	Vitamin E ↑ endocytosis of PLGA	353

Polysaccharides	Hyaluronic acid	n/a	n/a	Au	5, 10, 20, 50, 100 nm	0.01–0.25 mg mL^−1^	24 h	ARPE-19, NIH 3T3, CHO	↑ uptake of 50 and 100 nm Au NPs functionalized with HA	343
		n/a	n/a	Au	30–50 nm	0–18 μg mL^−1^	n/a	HepG2, NIH 3T3	MET-H-AuNPs ↑ apoptosis in HepG2; no impact on NPs uptake	344
		n/a	n/a	siRNA	50–80 nm	100 nM	12 h	A549, H69, MDA-MB468, Hep3B, B16F10	↑ siRNA silencing activity in presence of HA	346
		n/a	n/a	PEG–PLGA	250–350 nm	n/a	12 h	L929, MDA-MB-231, MCF-7, HepG2	↑ endocytosis of HA–PEG–PLGA micelles in MDA-MB-231 and MCF-7	345
	Mannose	n/a	n/a	Liposomes	1000 nm	1 mM	2 h	NR8383	↑ uptake of liposomes in the presence of mannose	348

### 6.1 Cytokines and other inflammatory molecules

Much attention has been given in recent years to investigate interactions between phagocytic cells and NPs.^22^ This is beneficial to better understand the behaviour of NPs in the human body in the presence of inflammatory molecules and their impact on phagocytic activity. Phagocytes are specialized cells of the innate immune system, which include monocytes, macrophages (mature monocytes), granulocytes (neutrophils and eosinophils) and dendritic cells. Their primary role is to eliminate foreign macromolecules/particles *via* macro- and microscale endocytic pathways and to degrade them by specific enzymes in lysosomes. As a response, phagocytes are able to secrete different inflammatory molecules, interferons, interleukins, and growth factors called cytokines, which stimulate a specific immune response by recruiting lymphocytes. Additionally, phagocytes are able to respond to different inflammatory stimuli from their local microenvironment.^291^

**Fig. 6:**
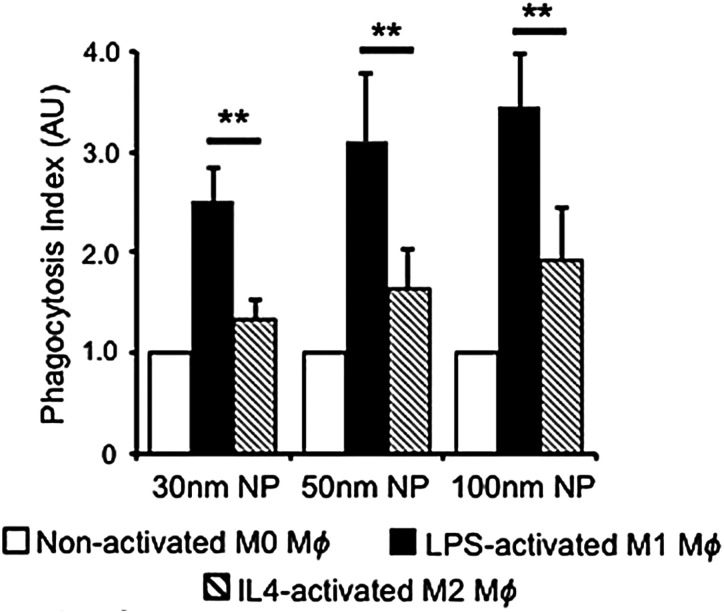
Increased uptake of different sized PS NPs in lipopolysaccharide-activated macrophages analyzed by flow cytometry and expressed as phagocytosis index (AU). Adapted with permission from Qie *et al.*^292^ Copyright 2016 Qie *et al*.

Inflammatory stimuli not only reprogram (polarize) macrophages into pro-inflammatory M1 phenotype or anti-inflammatory M2 phenotype but can also enhance NPs uptake. Hoppstädter *et al.*^22^ investigated endocytosis of SiO_2_ NPs (26 and 41 nm) and SiO_2_ microparticles (1.75 μm) by different macrophage sub-populations, while stimulating them with various molecules: lipopolysaccharide (LPS)/interferon (IFN)-γ to generate M1 phenotype and interleukin (IL)-10 to differentiate cells into M2 macrophages. NPs uptake was higher in M2-polarized macrophages compared to M1 cells in all macrophage subpopulations. Higher endocytic capacity in M2-polarized cells has been explained due to higher expression of endocytosis-facilitating scavenger and lectin receptors on the surface. Interestingly, the uptake of microparticles did not differ between M1 and M2 phenotypes. In contrast, Qie *et al.*^292^ observed increased uptake of differently sized PS NPs (30, 50, and 100 nm) by M1 primary bone-marrow derived macrophages, stimulated with LPS (Fig. 6) or LPS and IFN-γ.

According to the Food and Drug Administration (FDA), a novel nanomedicine, CYT-6091 (Aurimune™), constructed by binding recombinant human tumor necrosis factor alpha (rhTNF-α) to colloidal gold NPs, was tested in Phase 1 of the clinical trials in cancer patients.^293^ It has been demonstrated that colloidal gold-bound rhTNF-α successfully targets the tumors and can be administered at doses of rhTNF-α that were previously shown to be toxic to the patients. In the future, clinical studies will focus on combining CYT-6091 with approved chemotherapies for the systemic treatment of non-resectable cancers.^294^

Cytokines are not the only stimuli that can enhance NPs uptake by phagocytes. When NPs are exposed to biological fluids, formation of protein corona on NPs surface strongly influences their interaction with cells. For example, several serum proteins (immunoglobulins, albumin and fibrinogen) can act as ligands for macrophage receptors.^295,296^ Human monocytes (THP-1) were treated with the following cytokines to generate specific phenotypes: IFN-γ/LPS for M1 and IL-4/IL-13 for M2. Cellular uptake of SiO_2_ NPs (50, 100, 200, 500 and 1000 nm) was investigated with or without the presence of human serum in the cell culture medium. Uptake of 200–1000 nm NPs in the presence of serum was higher by M2 compared with M1 macrophages. The reason could be due to higher expression of receptors against protein corona ligands (fibrinogen, IgG and HDL) on M2 macrophages.^295^ In addition, higher uptake of various cargos other than NPs (*e.g.* HDL, LDL, and bacteria) in phagocytic cells when exposed to inflammatory stimuli (cytokines) has also been observed.^297–299^

There is no rule about which stimuli/NPs combination generally causes the highest uptake rate in phagocytes. We conclude that the uptake highly depends on cell type and NP physicochemical properties. A deeper knowledge of the interaction between NPs and phagocytes in the presence of inflammatory stimuli is of a great importance in nanomedicine as such NPs/drugs are or will be mainly applied in diseased patients. For instance, inflammatory stimuli can act as a NPs uptake accelerant to increase the delivery of a pharmaceutical substance to cancer cells.^300^

### 6.2 Co-exposure with different nanoparticles

With the development of nanotechnology, NPs have emerged as potential carriers for therapeutics. Several studies in recent years have pointed out that NPs can be internalized faster, while co-exposed due to activation of multiple parallel endocytic pathways, *i.e.*, synergistic effect.^23,24,134,301–304^ This suggests NP co-exposure as a potential approach to increase the intracellular accumulation of drug-loaded NPs. When exposing cells to multiple particles, cytotoxic effects should also be considered.

**Fig. 7:**
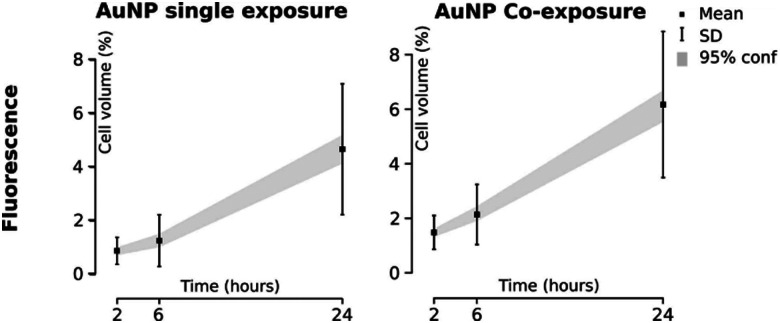
Quantification studies based on confocal laser scanning microscopy (CLSM) show a time-dependent increase in uptake of Au NPs in J774A.1 macrophages. The highest increase was observed in Au NPs co-exposure with Fe_3_O_4_ NPs. Adapted with permission from Vanhecke *et al.*^24^ Copyright 2017 Vanhecke *et al*.

Vanhecke *et al.* revealed synergistic effects of Au and Fe_3_O_4_ NPs co-exposure on murine macrophage cells (J774A.1).^24^ Live cell imaging with confocal microscopy, environmental scanning electron microscopy (SEM) and ICP-OES data showed that co-exposure accelerates uptake due to activation and crosstalk between different endocytic pathways (Fig. 7).

Tsugita *et al.* examined the effect of combined exposure of SiO_2_ and TiO_2_ NPs on murine bone-marrow-derived macrophages.^301^ This type of co-exposure synergistically induced the macrophage inflammatory response by use of distinct cellular mechanisms to cause cellular stress but does not reveal any effects on cellular NPs uptake.

Guo *et al.* examined synergistic oxidative effects of carbon black and Fe_3_O_4_ NPs co-exposure on human lung epithelial cells (A549) indicating intracellular redox reaction between both NPs.^23^ No significant difference was found in Fe_3_O_4_ NPs uptake between single- and co-exposure.

The synergistic effect of combined NPs exposure, such as silver and cadmium,^304^ SiO_2_ and arsenic,^303^ PS latex and poly-lactic-*co*-glycolic acid^302^ has also been investigated. These studies have focused mainly on synergistic cytotoxic effect, but no information is mentioned about their effect on cellular uptake.

### 6.3 Bio-inspired molecules

Nature has developed structurally and molecularly diverse substances, which can be used in combination with NPs (*e.g.* as a surface coating or co-exposure approaches) to achieve higher cellular uptake. Bio-inspired molecules have become increasingly interesting in science due to their ability to serve as alternative biocompatible drug delivery systems.^305^ In this section, various bio-inspired molecules found in the literature are discussed.

#### 6.3.1 Cell penetrating peptides

Selective membrane permeability controls the uptake of several molecules, including NPs and therapeutics to reach their sites of action inside the cells.^306^ It has been recently shown that cell-penetrating peptides (CPPs), a family of small 5–30 amino acid peptides, could be used to increase NPs internalization. According to their physicochemical properties, these peptides can be categorized into three main classes: cationic, amphipathic and hydrophobic.^307^ CPPs can pass through cell membranes *via* energy-independent penetration by forming interactions with negatively charged groups (*e.g.* inverted micelles or pore formation) or *via* energy-dependent endocytic pathways (*e.g.* micropinocytosis, caveolae- and clathrin-mediated endocytosis).^308^ The activation of a specific uptake pathway depends on the size and physicochemical properties of the CPPs.^309^ Adsorption of CPPs to NPs is driven by electrostatic and hydrophobic interactions, involving mainly arginine residues and the NPs surface.^27^

**Fig. 8:**
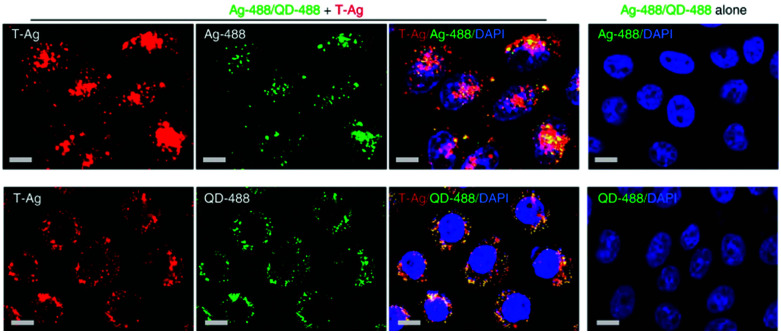
Ag NPs functionalized with cell-penetrating peptide TAT (T-Ag; red) enhanced cellular uptake of bystander NPs (Ag-488 and QD-488; green) in CHO cells. Representative CLSM images for Ag-488 (top panels) or QD-488 (bottom panels). Scale bar: 10 μm. Reproduced with permission from Wei *et al.*^195^ Copyright 2019 Wei *et al*.

NPs conjugates with CPPs could enhance NPs uptake and therapeutic effectiveness without causing significant cell damage. One of the first discovered CPPs was the cationic trans-activator of transcription peptide (TAT) from human immunodeficiency virus 1 (HIV-1).^310^ It has been demonstrated that TAT peptide is highly efficient in translocating different small molecules across the cell membrane.^311–313^ TAT peptide enhances the uptake of multiple bystander cargo (*e.g.* AgNPs, AuNPs, QDs, IONPs, BSA and dextran) through the macropinocytosis pathway in different cancer cell types (Fig. 8).^195,313^

Shortly after TAT-peptide discovery, a homeoprotein penetratin of fruit fly *Drosophila melanogaster* has also been shown to have the ability for transporting therapeutic molecules across the cell membrane.^314^ More than 100 peptides have been used since then to accelerate therapeutics delivery, such as siRNA,^315^ antisense oligomers,^316^ enzymes,^317^*etc.* into eukaryotic cells in preclinical and clinical trials.^307^

Several studies exhibited the potential of NPs–CPP conjugates to enhance therapeutics delivery to various cancer cells both *in vitro* and *in vivo*, such as brain glioma,^318^ breast cancer,^319^ hepatocarcinoma,^320^ lung cancer^321^ and others. Conjugation of PEG–poly(lactic-*co*-glycolic acid) (PEG–PGLA) NPs with Pep–TGN peptides resulted in 3.6-fold higher delivery of coumarin-6 into brain cells compared to unmodified NPs.^322^ Cellular uptake of different NPs–CPP conjugates into drug resistant cancer cells showed that CPPs uptake is cell type-dependent.^323^

CPPs in combination with NPs can also be used as imaging agents to follow the pharmacokinetics and pharmacodynamics of the therapeutics. Liu *et al.* demonstrated that arginine-rich CPPs significantly increase cellular uptake of QDs.^324^ Higher accumulation of SPIONs functionalized with (CPP)–polyarginine peptides in comparison to unconjugated SPIONs in bladder cancer cells has also been described.^325^

The potential of CPPs to increase cellular NPs uptake has been applied for inflammatory disease treatment in mice, *e.g.* delivery of anti-rheumatic drug methotrexate using lipid polymeric hybrid nanoparticles (LNPs) conjugated with stearic acid–octarginine, folate–PEG–PLGA and polyketal (PK3).^326^ Finally, yet importantly, Yan *et al.* demonstrated the potential of NPs–CPP conjugates in neurodegenerative disease treatment, *e.g.* TAT functionalized PLGA NPs showed 4.5-fold increase in intracellular accumulation of insulin in the brain.^327^

Although there is an increasing number of CPPs reported in the literature, none of the CPP-based treatments have been approved yet by the FDA. The reasons could be a lack of proteolytic stability, selectivity, and the issues with the endosomal release. However, many CPPs are used in (pre)clinical studies, showing promising results in treating diseases, such as cancer.^328,329^

In conclusion, NP functionalization with CPPs has a great potential to enhance delivery of therapeutics into cells without provoking any cell damage. Structural characteristics such as sequence length, amino acid composition, and chirality play an important role in CPPs uptake and should be investigated for future improvements of efficiency of NPs as drug delivery carriers. Furthermore, development towards protease stable and water soluble CPPs is highly desirable.

#### 6.3.2 Bacterial toxins

Natural pathogens, such as viruses and bacteria can be internalized into mammalian cells by sharing similar endocytic pathways with NPs.^330,331^ Internalization *via* a caveolae-dependent pathway is induced by specific ligands such as cholera toxin,^332^ simian virus-40,^333^ and bacteria.^334^ Microorganisms internalized *via* caveolae-dependent endocytosis can escape lysosomal degradation.^86^ This pathway is considered to be predominant also for endocytosis of some NPs (Section 2.4).^93^ By avoiding lysosomal degradation, such a mechanism could be beneficial for targeted NPs delivery.

By co-exposing cells to NPs and bacteria or their toxins and thus stimulating more endocytic pathways simultaneously, NPs uptake and therapeutics delivery can potentially be increased. Akin *et al.* co-exposed NPs and bacteria to deliver DNA-based therapeutics. A fluorescent or bioluminescent gene was loaded as a cargo on the NPs, which were carried on the bacteria. After exposure to different tumor cell lines, the cargo-carrying bacteria were phagocytosed. Bacterial toxins caused the disintegration of endosomal compartments and the cargo was released intracellularly from the NPs. This approach aimed to use bacteria as a carrier for various therapeutic peptides, antibodies or small molecule drugs.^335^

#### 6.3.3 Food supplements

NPs are widely applied not only in nanomedicine but also as additives in consumer products. Food supplements, such as vitamins, sugars, and amino acids can interact with NPs in many ways (*e.g.* by formation of protein/biomolecule corona around NPs) and alter the cellular response.^336^

Folic acid (FA) is the synthetic form of folate, a water-soluble B_9_ vitamin. It is a natural product in foods such as green vegetables (lettuce, spinach, and broccoli), legumes (lentils, peas, and beans), and fruits (oranges, and mangoes).^337^ Many cancer cells overexpress FA receptors on their surface. Functionalization of NPs with FA could enhance NPs uptake in cancer cells as a specific targeting approach.^338–340^ FA was conjugated to MSNs loaded with the chemotherapeutic drug topotecan and delivered to retinoblastoma cells. The FA-conjugated NPs exhibited higher uptake in retinoblastoma cells compared to that of non-conjugated NPs. The higher uptake was attributed to FA receptor-mediated endocytosis.^341^

**Fig. 9:**
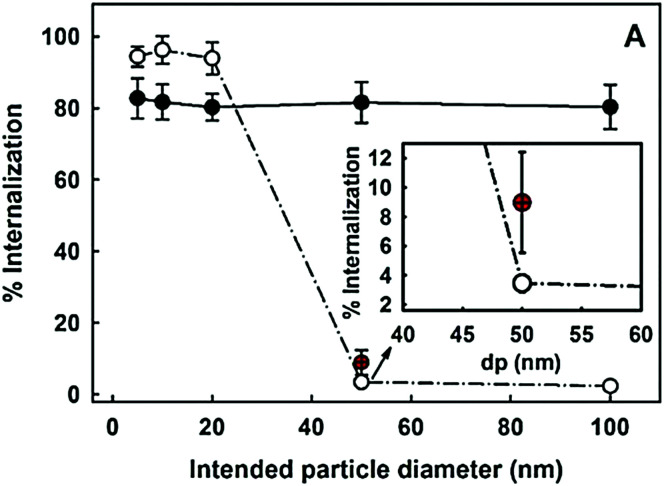
ICP-MS data showing increased uptake of Au NPs coated with (●) end-thiolated hyaluronic acid (HS–HA) and compared to (○) bare Au NPs in human retinal pigment epithelial cells. Adapted with permission from Elsevier.^343^ Copyright 2018 Elsevier.

Hyaluronic acid (HA) is a carbohydrate, naturally present in the extracellular matrix in all living organisms. It is one of the most hydrophilic molecules found in nature, responsible for tissue hydration and water transport.^342^ HA serves as a ligand for many cell-surface receptors and has been investigated as a drug delivery agent in combination with various NPs. Karakocak *et al.* demonstrated facilitated uptake of 5–100 nm AuNPs, coated with end-thiolated hyaluronate (HS–HA) compared to that of uncoated NPs (Fig. 9).^343^ They also illustrated that the presence of CD44 receptors on the cell surface can facilitate HS–HA–AuNPs uptake. HA-conjugated NPs have potential as drug delivery vectors for cancer cells overexpressing CD44 receptors. The increased uptake of other NPs, such as AuNPs,^344^ PEG–PLGA^345^ and siRNA^346^ functionalized with HA has also been demonstrated.

Mannose is a sugar monomer and a natural component of some plants (*e.g.* cranberries). Mannose-conjugated NPs can be used for targeted NPs delivery.^347^ Chono *et al.* exposed alveolar macrophages and NR8383 cells to liposomes, mannosylated with 4-aminophenyl-α-d-mannopyranoside. The differences in cellular uptake between mannosylated and non-mannosylated NPs were evaluated *in vitro* and *in vivo*. The uptake of mannosylated liposomes by both cell types was significantly greater compared to non-modified liposomes. The uptake mechanism of mannosylated liposomes by alveolar macrophages is believed to be mannose receptor-mediated.^348^

Vitamins are a group of essential organic compounds, which are required in nutrition for maintaining health, development and metabolic integrity. They function as co-enzymes, hormones, antioxidants, cell signaling mediators, growth regulators *etc.*^349^ In addition, some vitamins can enhance cellular NPs uptake. One of such vitamins is water-soluble vitamin B_12_ (*i.e.* cobalamin). Dietary products rich in vitamin B_12_ are fish, poultry, eggs and milk. B_12_ serves as a co-factor for enzymes, involved in amino acid synthesis. Its adequate supply is essential for red blood cell generation and optimal function of central nervous system.^350^ Russell-Jones *et al.* showed that conjugation of NPs (50 nm, 100 nm and 200 nm) with vitamin B_12_ highly enhanced their uptake into Caco-2 cells, an intestine cell line, compared to uncoated NPs.^351^ Wang *et al.* investigated the endocytosis and cytotoxicity of zinc oxide (ZnO) NPs and vitamin C co-exposure in gastric epithelial and neural stem cells.^352^ The presence of vitamin C increased intracellular concentration of Zn^2+^ ions and ZnO-associated cytotoxicity. Vitamin C created an acidic environment and caused the dissociation of ZnO into Zn^2+^ ions. By intracellular Zn^2+^ elevation, zinc homeostasis was disrupted, resulting in lysosomal and mitochondria damage and apoptosis.

Yin Win *et al.* showed that surface coating of PLGA NPs with vitamin E greatly improved adhesion to and cellular uptake into intestinal Caco-2 cells.^353^ In addition, it increased the ability of NPs to escape the cells *via* multi-drug resistance pumps. Since coating with vitamin E could increase NPs cellular uptake and inhibit P-glycoprotein-mediated drug transport, this mechanism could potentially improve the efficacy of oral NPs delivery.

A NPs-based therapeutic ND L02-s0201 (Nitto BioPharma Inc.) has been developed for the treatment of fibrosis of liver and other organs. It specifically targets hepatic stellate cells through the use of liposomes that are conjugated to vitamin A.^354^ Hepatic stellate cells will specifically take up vitamin A, so the drug is delivered to the stellate cells preferentially, but not to other liver cells. The specific targeting of stellate cells together with delivery of a siRNA that can downregulate collagen production has resulted in the reversal of cirrhosis and liver regeneration. The nanoformulation has already entered Phase 2 of clinical trials.

## 7. Decreasing NP endocytosis

The majority of studies investigated drug-loading capability of functionalized NPs in combination with different stimuli to improve NPs uptake into target cells. Another very important aspect is to investigate specific interactions of NPs with the microenvironment resulting in reduced cellular uptake. For instance, internalization of NPs loaded with therapeutics by cells of a mononuclear phagocyte system (MPS) decreases the treatment efficacy for other target cells/tissues. It is important to explore ways to make NPs invisible for the MPS, but still ensure highly efficient delivery and internalization of NPs into target cells.^356^ It has been shown that some pathogens are able to survive long periods in hosts without being detected and eliminated by the immune system.^357^ Therefore, a possibility exists to coat NPs with specific biomolecules that protect them from being phagocytosed by MPS and to prolong its circulation. The concept could also be used to decrease the uptake of unintentionally ingested or inhaled NPs. Table 3 represents an overview of the substances related to decreasing NPs uptake.

**Table 3:** Summary of main substances related to reduced NPs uptake

	Stimuli			Particles				Cell types	Summary of effects	Ref.
	Types	Concentration	Exp. time	Material	Size	Concentration	Exp. time			
**Inflammatory molecules**										
Cytokines	IL-4, IL-13	2–6 ng mL^−1^	48 h	Neisseria, *E.coli*, zymosan	n/a	100 per cell	2 h	Mouse peritoneal macrophages	IL-4 and IL-13 ↓ uptake of bacteria and zymosan *via* IL-4/IL-13 receptors	362
	IL-4	10 ng mL^−1^	48 h	YG™	1 μm	10^8^ mL^−1^	0.5 h	Primary BM mononuclear cells	IL-4 ↓ phagocytosis of YG beads in macrophages	364
		20 ng mL^−1^	48 h	B-lymphoma cells	n/a	10^6^ mL^−1^	n/a	Human macrophages	IL-4 ↓ phagocytosis of rituximab opsonized lymphoma cells by macrophages	365

**Co-exposure with different nanoparticles**										
Nano-particles	SiO_2_	10–250 μg mL^−1^	24–72 h	Fe_3_O_4_	20–30 nm	10–250 μg mL^−1^	24–72 h	A549	Effect on NPs uptake unknown; ↓ ROS and toxicity in co-exposure	367

**Surface modification**										
Proteins	HSA	100 μM	n/a	DHLA-QDs	5 nm	10 nM	2 h	HeLa	HSA ↓ DHLA-QDs endocytosis	418
	FBS	0, 10, 55%	n/a	SiO_2_–PEG–Tf	50 nm	50 μg mL^−1^	1–8 h	A549	FBS proteins ↓ TfR targeting for Tf-conjugated NPs	272
Polyethylene-glycol	Amino-PEG	10.000 g mol^−1^	n/a	PS	30, 50, 100 nm	10^13^–10^15^ mL^−1^	8–24 h	Primary BMDM	Amino-PEG coating ↓ PS NPs uptake	292
	PEG	n/a	n/a	FePt, Fe_3_O_4_	4–11 nm	5–10 nM	12 h	NIH/3T3	PEGylation ↓ uptake of the NPs for 10%	375
	Thiolated-PEG	50 PEG per nm^2^ Au	n/a	Au	15 nm	20 nM	1–24 h	A549	Thiolated-PEG ↓ uptake of Au NPs	128
Surface markers	CD47	n/a	n/a	PS	30, 50, 100 nm	10^13^–10^15^ mL^−1^	8–24 h	BMDM	↓ phagocytosis of PS NPs, coated with CD47	292
		21 μm^−2^ bead	n/a	Streptavidin–PS	100, 160, 1100, 3500 nm	n/a	3/4 h	THP-1	CD47 ↓ phagocytosis of streptavidin–PS by signalling through the phagocyte receptor SIRPα	398

**Bio-inspired molecules**										
Bacterial proteins	Yersinia (Yop)	400 μg mL^−1^	n/a	n/a	n/a	n/a	n/a	HeLa	YopE ↓ internalization of bacteria by ↓ PI3K/AKT pathway	419
		n/a	1 h	n/a	n/a	n/a	n/a	Murine BMDC	YopE, YopH and YopT ↓ uptake of bacteria by actin fibers destruction	408
		n/a	1 h	n/a	n/a	n/a	n/a	J774A.1	YopH ↓ internalization of bacteria *via* ↓ PI3K/AKT pathway	407
	Streptococcus (Sic)	10 μg mL^−1^	1 h	n/a	n/a	n/a	n/a	PMN	Sic ↓ phagocytosis of bacteria	409
Viral proteins	Herpes virus (K14)	n/a	n/a	n/a	n/a	n/a	n/a	MDM	K14 ↓ macrophage activation by interacting with human CD200R; unknown effect on uptake	420
Fatty acids	Oleic, stearic, α-linoleic	500 μM	n/a	ZnO	22 nm	4 μg mL^−1^	6 h	THP-1	FFA ↓ the NPs uptake and alter NPs–cell interactions	411

**Others**										
Chloroquine	n/a	20–400 μM	n/a	Albumin, lyposome, PS, silicon	14–264 nm	Albumin: 20 μg mL^−1^; liposomes: 200–400 μM; PS: 3 × 10^9^ mL^−1^; silicon: 6 × 10^7^ mL^−1^	3–6 h	J774A.1, RAW264.7, Kupffer cells MDA-MB-231, MIA PaCa-2, H358 lung cancer cells	Chloroquine ↓ NPs uptake *via* clathrin-mediated endocytosis by ↓ PICALM expression	179

### 7.1 Cytokines and other inflammatory molecules

Cytokine stimulation of macrophages,^297^ dendritic cells,^358,359^ fibroblasts^360^ and endothelial cells^361^ can regulate cellular endocytic mechanisms. An *in vitro* study investigated the uptake of the pathogenic bacteria *Neisseria meningitidis* in mouse peritoneal macrophages after IL-4 and IL-13 pretreatment.^362^ These cytokines are responsible for alternative activation of macrophages towards M2 anti-inflammatory phenotype.^363^ After 48 hours of IL-4 pretreatment, a remarkable reduction in *Neisseria meningitidis* uptake was reported. Reduction of uptake was also observed with *E. coli* and zymosan. Coincidently, IL-4 activation stimulated the secretion of pro-inflammatory cytokines by macrophages. Reduced phagocytosis of bacteria was due to inhibition of phagosome formation by down-regulation of PI3K activity.^362^ The inhibitory effect of IL-4 on uptake of 1 μm fluorescent beads^364^ and rituximab-opsonized lymphoma cells^365^ in macrophages have also been shown. We assume that decreased cellular uptake caused by IL-4 or IL-13 not only impacts uptake of bigger particles in the micrometer size but could also apply for NPs. Decreased uptake after cytokine stimulation has also been observed for dendritic cells. Patente *et al.* induced dendritic cell maturation by stimulation with the cytokines TNF-α and IFN-γ.^359^ While the immature cells expressed high endocytic capacity, the cytokine-stimulated mature dendritic cell showed a decrease in endocytic activity.

### 7.2 Co-exposure with different nanoparticles

In addition to many synergistic effects,^23,301,366^ few studies have demonstrated the antagonistic effect of NPs co-exposure. Rafieepour *et al.* investigated the *in vitro* toxicological effects of single and combined exposure of magnetite (Fe_3_O_4_) NPs and polymorphous silica NPs on human epithelial cell line A549.^367^ The cells were exposed to four different NP concentrations (10, 50, 100, and 250 μg mL^−1^) of both NP types simultaneously for 24 h and 72 h. The data obtained in this study showed that increasing the concentration and exposure time to Fe_3_O_4_ and SiO_2_ NPs individually increased toxic effects. In contrast, the effect of combined exposure to Fe_3_O_4_ and SiO_2_ NPs led to antagonistic interactions. Combined exposure resulted in decreased reactive oxygen species (ROS) production and reduced toxicity (*i.e.* antagonistic effect) in comparison to single exposures. The reason for antagonistic effect in combined exposures has been explained by the accumulation of intracellular proteins on the SiO_2_ surface, forming a protein corona. Fe_3_O_4_ could provoke the synthesis of cellular proteins, causing the formation of protein corona on the surface of silica NPs and thereby reducing its cytotoxic effect.^368,369^ This phenomenon needs to be investigated further and we assume that another mechanism might be involved in the antagonistic effect of the two NPs. Unfortunately, this study did not investigate the antagonistic effect of combined exposure on intracellular uptake of NPs.

### 7.3 Protein corona

NPs exposure to complex biological environment results in non-specific protein coating of particles, known as a “protein corona”.^370^ The corona consists of two components: (i) the inner firmly bound high-affinity proteins, known as “hard corona” and (ii) the outer loosely associated low-affinity proteins described as “soft corona”.^124,371^ “Hard” corona changes over time in terms of the amount of bound proteins, but not in composition.^372^ The majority of *in vitro* studies focus on the “hard” corona, as these proteins are strongly attached on the NPs and do not detach upon extensive washing.^373^ Phagocytes are able to recognize the proteins on the NPs surface and target them for internalization. The formation of a corona can reduce cellular uptake of functionalized NPs by shielding the ligands from binding to their receptors. As an example, the attachment of serum proteins on the surface of transferrin-functionalized SiO_2_ NPs resulted in a loss of its targeting capability.^272^ This “shielding” activity covers the active binding sites on the NPs and prevents the recognition by transferrin receptors. The composition of a protein corona is an important determinant of NPs fate and their cellular internalization. Various proteins, such as human serum albumin,^374,375^ fibrinogen,^375^ apolipoprotein,^376^ transferrin,^377^ and complement proteins^378^ were observed to adsorb onto the NPs.^379^ While some serum proteins contribute to decreased NPs endocytosis by shielding surface modification molecules on NPs, the binding of others may enhance the NPs uptake or modulate the endocytic mechanisms.^379,380^ This observation is supported by a multivariate model that used the protein corona fingerprint to predict NPs–cell association.^380^ Out of 64 tested serum proteins, 39 were classified as promoters of cell associations and 25 as inhibitors. The corona composition also varies depending on the nature of the biological fluids in which NPs are dispersed, such as human serum.^381^ Lesniak *et al.* demonstrated that protein corona of a very different nature is formed in the absence or presence of serum.^369^ In medium, supplemented with serum, the principal proteins that adsorb on NPs surface are immunoglobulins, complement proteins, and apolipoproteins, while under serum-free conditions mainly cytosolic proteins, components of the cytoskeleton, and membrane-associated proteins are adsorbed.^369^ When comparing *in vivo* and *in vitro* conditions, differences in NPs uptake should be considered due to variations in serum protein concentrations (*in vivo*, NPs in blood encounter much higher serum concentrations).^382^ Francia *et al.* reported lower uptake of 50 nm silica NPs into HeLa cells in the presence of a high amount of serum (62 mg mL^−1^) compared to that of NPs incubated with a five-times lower amount. The lower uptake could be related to differences in the corona composition. Additionally, since the corona biomolecules can mediate the uptake of NPs through recognition by specific receptors, it is likely that the free serum proteins compete for the same receptors and reduce the uptake of the corona–NPs complexes.^383^ When applying NPs in nanomedicine, one should consider the parameters that are affecting the protein corona formation, such as higher serum content, blood flow and higher serum complexity.^372^ A dynamic *in vivo* environment provides a continuous source of new proteins, resulting in more complex “hard” and “soft” coronae.^384^

### 7.4 Surface functionalization

#### 7.4.1 PEGylation

In the nanomedicine field, non-specific interactions of NPs with serum proteins should be avoided for certain applications, as it limits the ability of particles to reach the target site. NPs conjugation with polyethylene glycol (PEG) increases the NPs circulation in the bloodstream due to increasing hydrophilicity and reducing their opsonisation. The latter targets NPs for ingestion and clearance by phagocytes.^385^ PEGylation is thus desired for applications when NPs should be delivered to target cells different than the cells of MPS and avoid recognition and clearance by the immune cells.^375^ Several studies show that PEGylated NPs display increased *in vivo* blood circulation retention times as well as reduced cellular uptake by cells in comparison to bare NPs.^128,386–388^ PEGylation is effective in shielding the surface of NPs. It also causes a small increase in particle size, which may decrease the non-specific NPs uptake by cells of MPS, such as macrophages^292^ and fibroblasts.^375^ PEG coating reduced the adsorption of soluble proteins such as complements, glycosylated proteins, and lipoproteins on NPs and therefore contributed to the generic uptake reduction across macrophage populations.^292^ Other studies also demonstrated that PEGylation decreased NPs uptake by macrophages^292^ and fibroblasts.^375^ Additionally, exposure of A549 cells to citrate-stabilized and PEG-coated 15 nm AuNPs at the air–liquid interface resulted in decreased uptake of PEG-coated NPs.^128^ For this reason, PEGylation might not always be the best strategy to improve drug delivery. While NPs coated with PEG exhibit prolonged circulation time *in vivo* upon systemic injection, it might also favour poor NPs uptake by tumor cells.^390^ Despite this limitation, several PEGylated NPs have already been clinically approved. Doxil®, PEG functionalized liposomal doxorubicin, was the first approved (FDA, 1995) anti-cancer nanomedicine.^391^ After, other PEG-based formulations such as Promitil® (Lipomedix Pharmaceuticals),^392^ MM-302® (Merrimack Pharmaceuticals),^393^ BIND-014® (BIND Therapeutics),^394^ NC-6004 Nanoplatin® (Nanocarrier), Cornell dots (Wiesner Group), *etc.* have been approved by the FDA.

#### 7.4.2 Specific surface proteins

Conjugation of NPs with CD47 is another mechanism to decrease their cellular uptake. CD47 is an immunoglobulin-like protein, present on the cell surface of hematopoietic stem cells and the majority of cancer cells. It serves as a “don’t eat me” signal to prevent cells from being phagocytosed by macrophages.^29^ CD47 is a ligand for signal-regulatory protein α (SIRPα) receptor, expressed on macrophages and dendritic cells.^395,396^ Binding of CD47 to SIRPα causes phosphorylation of the cytoplasmic domain of the receptor and reduced actin–myosin contraction at the phagocytic synapses.^397^ Rodriguez *et al.* demonstrated decreased phagocytosis of streptavidin-coated PS beads (>100 nm) conjugated with synthetic human CD47 protein in THP-1 cells.^398^ Qie *et al.* investigated the effects of CD47 conjugation on NPs uptake by different macrophage phenotypes (non-activated M0 and activated M1 and M2 macrophages).^292^

**Fig. 10:**
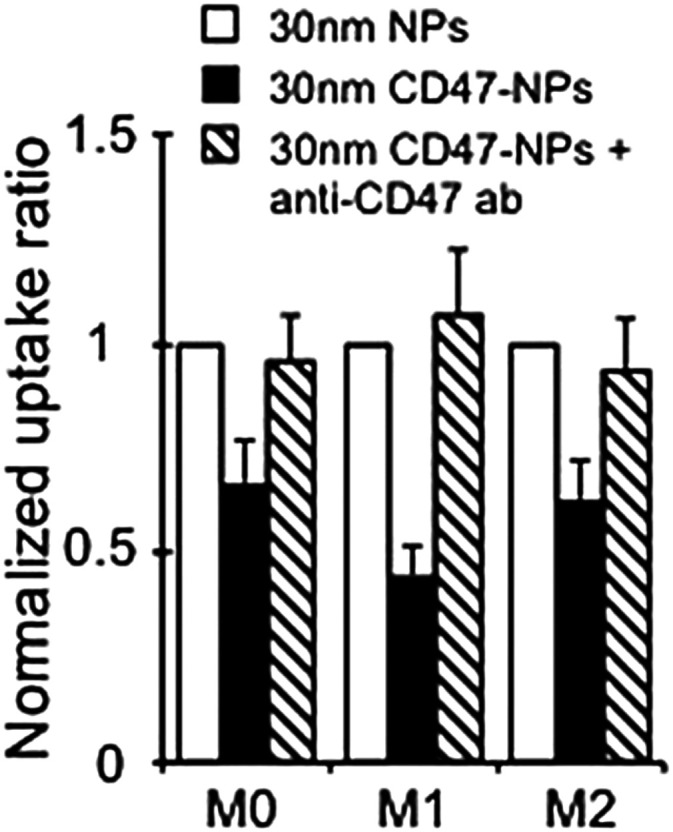
Surface modification of 30 nm PS NPs with CD47 protein reduced NPs uptake in macrophages. Adapted with permission from Qie *et al.*^292^ Copyright 2016 Qie *et al*.

Exposure of macrophages to NPs coated with CD47 showed a significant reduction in NPs uptake across all macrophage sub-populations (Fig. 10). Uptake reduction was mediated through CD47-SIRPα interaction. The most pronounced inhibition in NPs uptake was observed in M1 macrophages, probably due to their higher expression of thrombospondin-1 (TSP-1), which serves to strengthen SIRPα and CD47 interaction.^292,399^ Interestingly, co-exposure of macrophages to bare NPs and free CD47 also reduced NPs uptake, but to a lesser extent than CD47-conjugated NPs.^292^

### 7.5 Bio-inspired molecules

#### 7.5.1 Bacterial pathogens

Mammalian pathogens have evolved specific mechanisms to escape clearance by the host immune system. These natural mechanisms of phagocytosis inhibition can be exploited in nanomedicine for targeted drug delivery, where reducing phagocyte-mediated nanocarrier clearance is desired.

Pathogenic food-borne *Yersinia* is able to resist phagocytosis in macrophages. The reason for this is plasmid-encoded Yop proteins (*i.e. Yersinia* outer proteins).^400^ Expression of Yop effector proteins is induced upon contact with eukaryotic target cells (*e.g.*, macrophages), followed by channel opening and Yops injection into the cytosol.^401,402^ Several Yop proteins have been identified so far (YopH, YopE, YopM, YopK, and YpkA)^403–405,419^ and some share homology with eukaryotic proteins.^401^ Tyrosine phosphatase YopH is highly involved in the disruption of Fc receptor-mediated phagocytosis. It removes phosphate groups from the tyrosine-phosphorylated proteins, which are required for phagocytic invagination.^406^ Thus, YopH reduces the activation of the Akt pathway by blocking the integrin receptor-mediated activation of the PI3K signalling.^407^ The anti-phagocytic effect of Yersinia on macrophages and dendritic cells has also been demonstrated by other studies.^408^

Other pathogens, such as *Streptococcus* spp. are able to avoid host immune system by secreting specific molecules, which affect cytoskeleton function and inhibit phagocytosis. Many distinct *Streptococcus* serotypes have been identified, which differ in anti-phagocytic surface proteins (M proteins). Streptococcal inhibitor of complement (Sic) is a protein secreted by serotype M1. It has been shown that Sic binds with ezrin and moesin, two human proteins that functionally link the cytoskeleton to the plasma membrane. This data suggest that Sic together with other proteins decreases bacterial internalization and enhances its survival by enabling the pathogen to escape phagocytosis.^409^ Our idea in this context is to explore the usage of such bio-inspired proteins from bacteria in combination with various NPs to reduce their uptake.

### 7.6 Food supplements

Some food supplements can have an inhibitory (*i.e.*, antagonistic) effect on NPs uptake and subsequent cellular response.

Free fatty acids (FFA) are ubiquitous biological molecules, present in vegetable oils, avocado, various nuts, meat and milk products.^410^ After ingestion, the consumed FFA can interact with NPs. Jiang *et al.* investigated the interactions between ZnO NPs and saturated or unsaturated FFA. They observed significant reduction of intracellular Zn ions in THP-1 macrophages in the presence of stearic acid, oleic acid and α-linoleic acid. The reduced Zn uptake could be related to the altered NPs–cell interactions in the presence of FFA.^411^

Due to continuous exposure to multiple NPs and food supplements in our daily life *via* inhalation and ingestion, it is important to consider the potential interference of food supplements when assessing the biological effects of NPs.

### 7.7 Others

#### 7.7.1 Chloroquine

During the corona virus (COVID-19) outbreak, several attempts have been made to find a cure for this rapidly spreading disease. Among many others, chloroquine (known as an antimalarial drug)^412^ has been considered as one of the promising drug candidates for the treatment of COVID-19. It has been proposed that chloroquine might have an *in vitro* antiviral activity against SARS-CoV-2.^413^ The mechanism involves chloroquine-induced suppression of phosphatidylinositol binding clathrin assembly protein (PICALM), which might prevent endocytosis of SARS-CoV-2 virus and thus hamper its replication within cells. There is still no consensus regarding the application of the drug to treat coronavirus disease as some studies demonstrated no evidence of its efficacy.^414^ Despite these discrepancies, chloroquine still has a potential to be used in combination with NPs and reduce their uptake into non-target cells, such as macrophages.^179,415^ Wolfram *et al.* showed that chloroquine decreases the accumulation of albumin, PS NPs and nanoliposomes of various sizes (14–264 nm) and charge in macrophages.^179^ Pelt *et al.* found that chloroquine reduces the NPs uptake and accumulation by liver resident macrophages, leading to increased delivery of NPs into cancer cells.^415^ Mechanistic studies revealed that chloroquine indeed inhibits CME^416^ by reducing expression of PICALM (Table 1).^179^ In addition, chloroquine prevents lysosomal acidification and thus inhibiting its fusion with endocytic vesicles.^417^ It has been suggested that chloroquine most probably interferes with upstream endocytic trafficking, thereby blocking effective cargo transport.^415^

## 8. Conclusion

The use of NPs in nanomedicine for drug delivery and imaging is rapidly increasing. There is a high interest in development of novel delivery strategies, new technologies, new treatment approaches and research of novel NPs-based drug candidates. For biomedical applications, it is crucial to understand their interactions with different cell types as well as their behaviour in the *in vivo* system in the presence of various molecules. In recent years the knowledge about cell–NPs interaction, endocytosis and intracellular trafficking has largely increased. It is well known that the uptake of smaller NPs is mainly mediated *via* clathrin- and caveolae-mediated endocytosis, whereas bigger NPs and agglomerates enter the cells *via* macropinocytosis and phagocytosis. Based on the reviewed literature, there is still a lack of information regarding the NP type-specific endocytic mechanisms. In addition, the importance of clathrin-/caveolae-independent endocytosis needs to be explored in more detail. The main issue is the inconsistency between the existing results, which is a reflection of the complex nano–bio interactions as well as of the very different physico-chemical properties of different NP types. The choice of the optimal experiment is relevant, including the choice of the cell type, the introduction of more selective endocytic inhibitors such as dynasore, siRNAs and mutant proteins, as well as precise quantification of intracellular NP numbers and their subcellular localization. In an experimental set-up, a combination of different approaches for the assessment of NPs uptake and activation of specific endocytic pathways is highly recommended as most of inhibitors affect simultaneously different endocytic processes and may be cytotoxic. In addition, the advances in imaging techniques for sub-cellular analysis can provide new insights on the nano–bio interactions that occur at the cell surface and in subcellular compartments. Furthermore, CLEM and elemental analysis, flow cytometry and stereology became important tools for the accurate estimation of the number of internalized NPs (cellular dose). For the assessment of NPs–cell association as well as reliable quantification of the intracellular NP number it is recommended to combine different methods yielding complementary outcomes.

Despite many successful approaches used *in vitro* for delivery of NPs-based drugs into the cells, the *in vivo* trials have not yet proven to be successful, due to the insufficient amount/number of NPs reaching targeted cells. The major hurdles that NPs face upon *in vivo* administration are the protein opsonization, uptake by the MPS system and/or the failure of delivery and penetration into the target tissue. In order to improve their delivery, NP co-exposure or functionalization with various molecules has been proposed as a tool to overcome the issue of low targetability. For example, pro-inflammatory stimuli have great potential to increase uptake of many different NPs but they mainly work specifically on immune cells. Other bio-inspired molecules, such as CPPs, bacterial toxins, and food supplements have shown that it is possible to increase NPs uptake *in vitro* and have a potential to be explored further *in vivo*. In the last ten years, more than 400 nano-formulations have already entered the clinical phases. Most of them are currently in the clinical Phases 1 and 2 for the treatment of various types of cancer, proving the growing interest of the biomedical community in using NPs-based approaches for targeted drug delivery. Overall, the literature on molecules that decrease NPs uptake is, as of now, very scarce. Reduction of NPs clearance by MPS upon their *in vivo* administration is an important aspect in nanomedicine with the goal of improving targeted drug delivery. For example, NPs surface coating with PEG or CD47 can prevent NPs from being recognized by cell receptors. Thus, NPs avoid phagocytic clearance, and their circulation time within the human body can be prolonged. Additionally, some bacterial pathogens are capable of using natural mechanisms (*e.g.*, toxin release) to escape the endocytosis and survive longer in the host. Such approaches can be exploited further in combination with NPs. It is worth noting that when in contact with biological fluids, biomolecules present in the *in vivo* environment adsorb onto the NPs and form a protein corona. The corona formation alters the native properties and desired functionality of the NPs and thus modifies NPs uptake. Finally, food supplements and synthetic medications can interfere with NPs and reduce their uptake. Biological interactions are generally overlooked when designing NPs-based drug delivery systems. For this reason, it is important to consider the possible interactions between these substances and NPs to achieve efficient delivery to the target side. The increasing research interest in endocytic mechanisms can be merged with the chemical synthesis field to develop new NPs, which can be combined with nature-inspired substances, to improve physico-biochemical features for NPs delivery.

## 9. Future perspectives

The future perspectives in the field of improved targeting strategies in nanomedicine rely on the emergence of new tools capable of overcoming remaining knowledge gaps associated with the complex nano–bio interactions and the endocytic processes. The newly developed advanced tools should enable accurate discrimination between different endocytic pathways and an efficient quantification and intracellular localization of NPs. The introduction of CRISPR–Cas technology to specifically target key components of endocytic machinery presents a powerful tool that can be used to gain deeper insight into NPs uptake mechanisms and regulation of the pathways/processes. When applying NPs for biomedical purposes *in vivo*, the limitations lie in the fact that several factors are frequently overlooked in *in vitro* studies. Careful consideration of potential interactions of NPs with substances present in a realistic physiological microenvironment is essential for enhancing *in vivo* NPs delivery and for understanding the underlying mechanisms. Additionally, it would be of a great value to create a library of all potential molecules (natural and synthetic) that may have an impact, either stimulative or debilitating on NPs uptake. Examples of substances that could have a positive effect on the NPs uptake are inflammatory molecules, vitamins, polysaccharides, cell penetrating peptides and toxins. Encouraging preliminary results call for further investigation of these molecules. Ultimately, awareness and profound understanding of nano–bio interactions and how they drive cellular internalization is critical for successful implementation of NPs in nanomedical applications.

## Abbreviations

Au: Gold
BM: Bone marrow
CCP: Clathrin coated pit
CCVs: Clathrin coated vesicles
CLICs: Clathrin-independent carriers
CLSM: Confocal laser scanning microscopy
CME: Clathrin-mediated endocytosis
CPP: Cell-penentrating peptide
CTxB: Cholera toxin B subunit
EEA1: Early endosome antigen 1
EGF: Epidermal growth factor
FA: Folic acid
FcR: Fc receptor
Fe_3_O_4_: Iron oxide
FEME: Fast-endophilin-mediated endocytosis
FITC: Fluorescein isothiocyanate
GEEC: Glycosylphosphatidylinositol-anchored proteins-enriched early endosomal compartment
GPI: Glycosylphosphatidylinositol
GTP: Guanosine triphosphate
HA: Hyaluronic acid
HS: Human serum
ICP: Inductively coupled plasma
IFN: Interferon
IL: Interleukin
IONPs: Iron oxide nanoparticles
LDL: Low-density lipoprotein
LNP: Lipid nanoparticle
LPS: Lipopolysaccharide
MPS: Mononuclear phagocyte system
MSN: Mesoporous silica nanoparticles
NP: Nanoparticle
PEG: Poly(ethylene glycol)
PI3K: Phosphoinositide 3-kinase
PICALM: Phosphatidylinositol binding clathrin assembly protein
PLGA: Poly(lactic-*co*-glycolic acid)
PS: Polystyrene
PVA: Polyvinyl alcohol
QD: Quantum dot
RME: Receptor-mediated endocytosis
SiO_2_: Silica
SPIO: Superparamagnetic iron oxide
TiO_2_: Titanium dioxide

## Conflicts of interest

There are no conflicts to declare.
